# Combining Nanofiltration and Adsorbents to Achieve Effective PFAS Removal in Wastewater Effluent

**DOI:** 10.1021/acs.est.5c17011

**Published:** 2026-04-10

**Authors:** Aron M. Griffin, Jaidyn Elbers, Christopher Bellona, Timothy J. Strathmann

**Affiliations:** 3557 Colorado School of Mines, Department of Civil and Environmental Engineering, Golden, Colorado 80401, United States

**Keywords:** treatment train, granular activated carbon, anion exchange resins, perfluoroalkyl acids, nanofiltration

## Abstract

Adsorption of per- and polyfluoroalkyl substances (PFAS) by granular activated carbon (GAC) and ion exchange resins (IX) is negatively impacted by elevated concentrations of effluent organic matter (EfOM) and other background water constituents (e.g., coadsorbing inorganic ions) in complex matrices such as wastewater effluent. Here, we evaluated a hybrid system comprised of nanofiltration (NF) as an initial treatment step to reduce concentrations of PFAS, EfOM, and select inorganic ions, followed by either GAC or IX treatment of membrane permeate. A pilot membrane system utilizing a loose nanofilter (NF270) was operated continuously for >45 days, treating wastewater effluent with PFAS periodically added to the feed to evaluate rejection in high recovery (90%) batch experiments. Experimental results demonstrated >92% rejection of C ≥ 4 perfluoroalkyl acids (PFAAs), >98% rejection of hexafluoropropylene oxide dimer acid (Gen-X), and lower rejection (63–92%) of ultrashort chain PFAS and perfluorobutane sulfonamide (FBSA). Without NF pretreatment of wastewater effluent, rapid small scale column tests (RSSCTs) of adsorbents showed that PFAS maximum contaminant level (MCL) criteria were exceeded within 81 bed volumes (BVs) for GAC and 9,000 BVs for IX. In comparison, GAC treated >5,000 BVs of permeate from the NF experiments before exceeding MCLs, while IX-treated NF permeate never exceeded MCLs for the duration of the experiment (450,000 BVs). The proposed NF-adsorbent treatment train represents a promising strategy for PFAS removal from complex wastewater matrices, preventing point source discharges into the environment.

## Graphical abstract

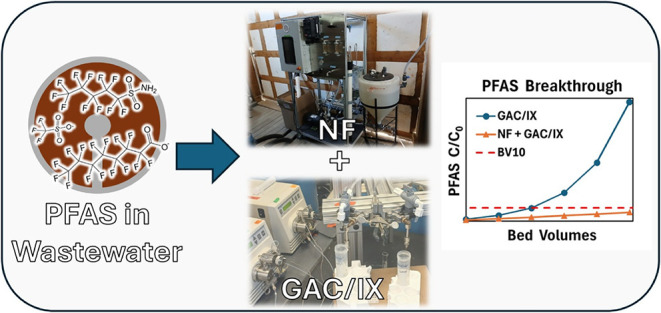

## 1 Introduction

Per and polyfluoroalkyl substances (PFAS) are ubiquitous in the environment due to their widespread use and the recalcitrance of carbon–fluorine bonds.^1^ Numerous human health impacts have been attributed to PFAS exposure, leading the U.S. Environmental Protection Agency (EPA) to issue drinking water standards and ambient water quality recommendations for several PFAS.^2^ Proposed drinking water maximum contaminant levels (MCLs) include a limit of 4 ng/L each for perfluorooctanoic acid (PFOA) and perfluorooctanesulfonic acid (PFOS); 10 ng/L each for perfluorohexanesulfonic acid (PFHxS), perfluorononanoic acid (PFNA), and hexafluoropropylene oxide-dimer acid (Gen-X); and a hazard index where the sum of the concentrations of PFHxS, PFNA, and Gen-X normalized to their respective MCL values and perfluorobutanesulfonic acid (PFBS) normalized to a value of 2,000 ng/L cannot exceed one. The EPA recently announced its intent to rescind and reconsider the MCLs for PFHxS, PFNA, and Gen-X and the hazard index, but these MCLs were still considered in this work as conservative treatment goals.^3^ To minimize further environmental contamination and protect drinking water sources, it is critical to treat point sources of PFAS pollution, including complex waste streams such as effluents from industrial and municipal wastewater treatment plants (WWTPs), which have been shown to be significant vectors of PFAS contamination into the environment.^4–7^

Municipal WWTPs do not actively add PFAS during treatment but receive PFAS-contaminated waste streams from both residential and industrial sources.^4,7^ Residential sources of PFAS include apparel, carpeting, personal care products, cleaning chemicals, and paper products.^7^ These products are disposed of in municipal landfills, where leachate is then sent to municipal WWTPs, or directly discharged into municipal sewer systems. Lin et al. found summed concentrations of terminal perfluoroalkyl acids (PFAAs) up to 80 ng/L and precursor concentrations up to 850 ng/L in residential wastewater, representing 26–98% of the total precursor loading at the three WWTPs evaluated.^7^ Municipal WWTPs also treat wastewater from car washes, laundry facilities, semiconductor manufacturing, electroplating, and other industries that can contain elevated PFAS concentrations.^5–9^ For example, effluent from semiconductor manufacturing facilities has been shown to contain up to μg/L concentrations of PFAS along with additional organic micropollutants, organic solvents, nanoparticles, and polymers that complicate treatment.^5,8^ Conventional physical and biological wastewater treatment technologies are not effective for PFAS removal, and concentrations of terminal PFAAs can increase in WWTPs due to biotransformation of precursor compounds, resulting in the presence of significant concentrations of PFAS in treated wastewater effluent.^4^

Adsorbents including granular activated carbon (GAC) and ion exchange resins (IX) have been successfully implemented for PFAS removal from groundwater and drinking water,^10,11^ but elevated concentrations of dissolved organic carbon (DOC) present in the form of effluent organic matter (EfOM) and inorganic ions characteristic of wastewater matrices can negatively impact PFAS adsorption processes, leading to accelerated breakthrough in fixed bed adsorbers.^12–14^ EfOM can directly compete for or block PFAS adsorption sites in GAC and IX, while inorganic ions can disrupt electrostatic interactions between PFAS and IX.^10,14^ Frequent media replacement results in increased operational complexity, cost, and waste production, limiting the feasibility of adsorbent use for PFAS removal in wastewater. Reverse osmosis (RO) or nanofiltration (NF) membranes can be applied, as an alternative to conventional adsorbents, to remove PFAS from complex wastewater streams, in combination with destructive treatment technologies to mineralize PFAS in membrane concentrate. RO provides near complete PFAS rejection, but elevated concentrations of nontarget solutes present in wastewater increase energy requirements and limit water recovery. Loose NF membranes with larger effective pore sizes (or molecular weight cutoffs (MWCO)) than RO can provide significant rejection of PFAS, especially longer chain compounds (>95% rejection), with lower required energy and propensity for fouling or scaling than RO.^9,11,15^ However, residual PFAS may be present in NF permeate at concentrations exceeding EPA MCLs, limiting the standalone use of NF in certain treatment applications (e.g., wastewater reuse and strict industrial treatment goals). Moreover, the majority of studies evaluating PFAS rejection by NF and RO membranes have been conducted over short durations in relatively clean water matrices; therefore, the impact of organic fouling and inorganic scaling on PFAS rejection is not well understood.^16,17^

To address the limitations of each respective treatment technology, this contribution evaluated a treatment train consisting of a loose NF membrane to provide initial rejection of PFAS and matrix constituents, including EfOM and inorganic ions that inhibit adsorption, followed by either GAC or IX treatment of NF permeate for further PFAS removal. A pilot-scale NF system outfitted with a Filmtec NF270 membrane was operated continuously to treat tertiary wastewater effluent for >45 days. A mixture of PFAS was spiked into the NF feed at intervals to evaluate PFAS rejection in high recovery (90%) batch experiments and determine the impact of continuous membrane exposure to wastewater on PFAS rejection. The mix of PFAS examined included long, short, and ultrashort chain PFAAs, Gen-X, and a sulfonamide precursor. Rapid small scale column tests (RSSCTs) then compared PFAS adsorption by GAC (Calgon Filtersorb 400) and PFAS-selective IX (Calgon Calres2301) in wastewater effluent with and without NF pretreatment. Additional RSSCTs were performed where PFAS were spiked into the NF permeate to specifically assess how membrane removal of nontarget constituents (e.g., EfOM, inorganic ions) affects PFAS adsorption. Findings from this work advance the understanding of PFAS rejection by NF membranes in wastewater, PFAS adsorption by GAC and IX in varied water matrices, and removal of ultrashort PFAS by NF and adsorbents and demonstrate the potential of a combined membrane-adsorbent treatment train for meeting strict PFAS limits when treating wastewater and other solute-rich matrices (e.g., landfill leachate, heavily aqueous film forming foam (AFFF)-impacted groundwater).

## 2 Materials and Methods

### 2.1 Membrane and Adsorbents Evaluated

This study evaluated the Filmtec NF270 membrane, Calgon F400 GAC, and Calres2301 IX. These are considered to be representative loose NF, GAC, and IX, respectively, that have been proven effective for PFAS treatment.^18–20^ The NF270 membrane was chosen instead of tighter NF or RO membranes due to its ability to reject PFAS with lower required energy and lower propensity for biofouling or scaling.^15^ The membrane also rejects nontarget ions to a lesser degree, limiting the buildup of salt in the membrane reject stream. Detailed characteristics of the membrane and adsorbents are provided in Tables S1–S3 in the Supporting Information (SI).

### 2.2 Tertiary Treated Wastewater Matrix

The tertiary treated wastewater effluent used in the NF and adsorbent experiments was supplied by a sequencing membrane bioreactor that treats domestic wastewater from student housing at the Colorado School of Mines. The system consists of two parallel 4500 gallon activated sludge bioreactors, followed by two membrane tanks containing PURON hollow fiber ultrafiltration membranes (0.05 μm pore size). Additional details of the wastewater treatment system are provided elsewhere.^21^ However, since the wastewater effluent did not contain appreciable concentrations of PFAS, a mixture of PFAS (Table 1) was spiked into the feedwater for the batch NF and adsorbent experiments. The concentrations of spiked PFAS were chosen to be representative of recently measured PFAS concentrations in industrial wastewater matrices (e.g., semiconductor fabrication).^5,9^ Additional structural information for the PFAS evaluated in this study is presented in Table S4 in the SI. Water quality parameters for the wastewater effluent used in the RSSCTs are provided in Table 1, and additional wastewater effluent parameters measured throughout the duration of the NF pilot experiment are provided in Table S5 in the SI.

**Table 1:** Characteristics of the Wastewater Effluent Matrix and Spiked PFAS Concentrations

Wastewater effluent matrix		PFAS spike		
Parameter	Concentration (mg/L)	Compound	MW (g/mol)	Concentration (ng/L)
Total Organic Carbon	4.23 ± 0.16	TFA	113	10,000
Total Nitrogen	6.02 ± 0.13	PFPrA	163	10,000
Cl^–^	75.7 ± 0.2	PFBA	213	500
NO_2_^–^	6.35 ± 2.37	PFPeA	263	500
NO_3_^–^	14.7 ± 0.3	PFHxA	313	500
PO_4_^3–^	13.4 ± 0.1	PFOA	413	500
SO_4_^2–^	130 ± 1	PFNA	463	500
Ca^2+^	37.3 ± 0.1	TFMS	149	500
K^+^	14.1 ± 0.1	PFBS	299	500
Mg^2+^	8.98 ± 0.04	PFHxS	399	500
Na^+^	53.2 ± 0.2	PFOS	499	500
Si^+^	2.91 ± 0.03	FBSA	298	500

### 2.3 Pilot Nanofiltration System

An automated pilot membrane system was used for the NF experiments. The system contained a single 2540 NF270 module and was continuously operated under a constant flux of 25 L per square meter per hour (LMH), a permeate flow rate of 1.1 L per minute, and a single pass recovery of 15% (calculations described in Section 2.6) for >45 days. Operation was facilitated by a LabVIEW control system with online flow, pressure, conductivity, and pH sensors. Under normal operation, the system treated wastewater effluent without the addition of PFAS in a flow-through configuration, where the NF permeate and concentrate were both discharged. This exposed the NF membrane to a continuous source of wastewater effluent with elevated concentrations of EfOM and select inorganic ions (Table 1 and Table S5 in the SI) and facilitated investigation of the impact of membrane fouling and scaling on PFAS rejection. On days 1, 15, and 30 of operation, duplicate batch experiments were performed where the mixture of PFAS described in Table 1 was spiked into the NF feed tank. The supply of wastewater effluent to the NF feed tank was discontinued, PFAS were spiked into the feed tank, and the NF permeate and concentrate were both recycled to the feed tank for 15 min to provide complete mixing. After 15 min, NF concentrate recycling continued, but the permeate was discharged until an effective water recovery of 90% was achieved (see Section 2.6). NF feed and permeate samples were collected at the end of recirculation (15% effective and single pass recovery) and at 25, 50, 75, 85, and 90% effective recovery set points (see Section 2.6). Permeate from the batch experiments was also collected and stored for use in subsequent adsorbent experiments (Section 2.4).

### 2.4 Rapid Small Scale Column Testing

RSSCTs were performed using the constant diffusivity approach to evaluate the performance of GAC and IX for the adsorption of PFAS.^22^ RSSCTs were chosen as they allowed for the evaluation of GAC and IX treatment of several feedwater sources in significantly less time and with less water than pilot-scale tests with full size media. Moreover, breakthrough data gathered through RSSCTs have been shown to be representative of pilot performance, and RSSCTs have been widely adopted for the evaluation of PFAS removal by GAC and IX.^13,22,23^ Three RSSCT feedwaters were evaluated: 1) wastewater effluent spiked with PFAS, 2) NF permeate from the treatment of wastewater effluent spiked with PFAS (treatment train), and 3) NF permeate spiked with PFAS after the treatment of wastewater effluent (PFAS-spiked permeate). The order of PFAS addition and pretreatment for each feedwater is shown in Figure 1. The third feedwater was included to assess the effects of reduced EfOM and other nontarget water constituent concentrations in NF permeate on adsorbent performance while maintaining initial PFAS concentrations comparable to the first feedwater. RSSCTs for each combination of feedwater and adsorbent were performed in duplicate, and PFAS were spiked in each feedwater at the composition and concentrations described in Table 1. The RSSCTs were performed using Waters 515 high performance liquid chromatography pumps and stainless-steel columns with an inner diameter of 0.43 cm. A stainless-steel frit with a 40 μm pore size was placed at both ends of the column to retain media. GAC RSSCTs were conducted with a 20 min simulated empty bed contact time (EBCT) and a4 gallons per minute per square foot (gpm/ft^2^) hydraulic loading rate (HLR), while IX RSSCTs were conducted with a 2 min simulated EBCT and 9.25 gpm/ft^2^ HLR. GAC and IX media were ground and sieved to average effective diameters of 58 and 68 μm, respectively. Additional details on RSSCT operation can be found in Table S6 in the SI.

**Figure 1:**
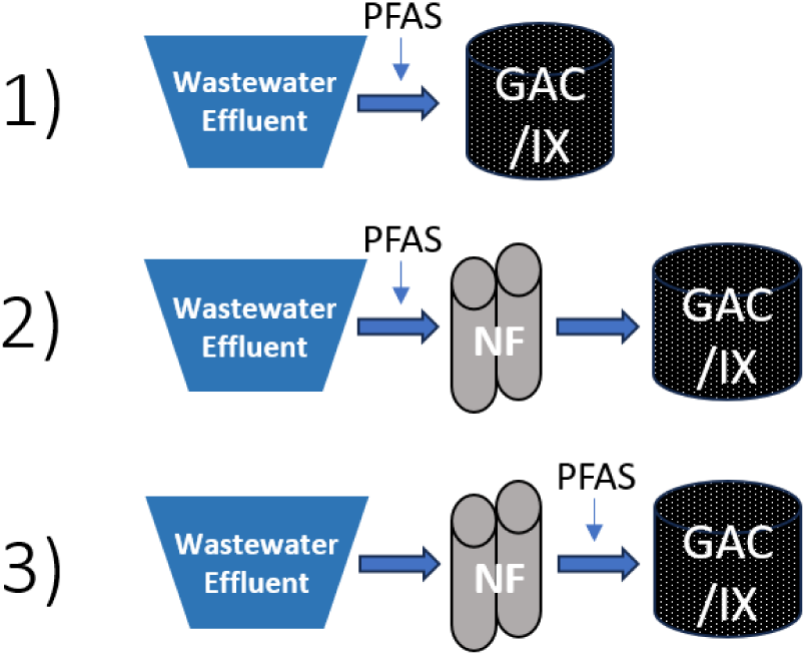
Schematic of the three RSSCT feed waters evaluated: 1) wastewater effluent spiked with PFAS, 2) NF permeate from the treatment of wastewater effluent spiked with PFAS (treatment train), and 3) NF permeate spiked with PFAS after the treatment of wastewater effluent (PFAS-spiked permeate).

### 2.5 Analysis Methods

PFAS were quantified by a SCIEX QTRAP 5500 using two separate methods. For ultrashort chain PFAS (trifluoromethanesulfonic acid (TFMS), trifluoroacetic acid (TFA), and perfluoropropanoic acid (PFPrA), a Shodex RSpak JJ-50 2D column was used with an isocratic eluent method consisting of 80% acetonitrile and 20% 50 mM ammonium acetate in water adjusted to pH 9 using ammonium hydroxide.^24^ All other PFAS were analyzed using a Phenomenex C18 column and a gradient method with eluents of 20 mM ammonium acetate and methanol.^25^ All eluents were Optima-LC-MS grade. Additional details of these methods and methods for general water quality analysis can be found in Section S5 of the SI.

### 2.6 System Performance Calculations

Membrane performance was characterized by both single pass and effective rejection and recovery. Single pass rejection (Rej_SP_) was calculated based on the solute’s instantaneous feed concentration (C_F_; i.e., the solute concentration in the NF feed tank at the time of measurement) and instantaneous permeate (C_P_) concentration:

1 $$Rej_{SP}=\left(1-\frac{C_{P}}{C_{F}}\right)\times 100$$

whereas effective rejection (Rej_Eff_) compared C_P_ to the solute’s concentration in the original feedwater (C_F‑Orig_) prior to batch operation:

2 $$Rej_{Eff}=\left(1-\frac{C_{P}}{C_{F‐Orig}}\right)\times 100$$

Single pass recovery (Rec_SP_) was calculated from the instantaneous feed (Q_F_) and permeate (Q_P_) flow rates:

3 $$Rec_{SP}=\frac{Q_{P}}{Q_{F}}\times 100$$

while effective recovery compared the amount of total permeate volume produced (V_P‑Total_) to the original volume of feedwater used in the batch experiment (V_F‑Batch_):

4 $$Rec_{Eff}=\frac{V_{P‐Total}}{V_{F‐Batch}}\times 100$$

Unless specifically noted, the recovery discussed in this work refers to effective recovery. Effective rejection values at 15, 25, 50, 75, 85, and 90% effective recoveries were combined into an overall batch rejection value by using the Riemann midpoint approximation. This compares the concentration in the original feed to the time-weighted average concentration of the permeate samples collected during batch operation.

Adsorbent performance was evaluated in terms of bed volumes (BVs) treated before breakthrough in RSSCTs. BVs were calculated by comparing the volume of water treated to the volume of the adsorbent bed.

5 $$BV=\frac{\mathrm{Flow}\;\mathrm{Rate}\times \mathrm{Time}}{\mathrm{Volume}\;\mathrm{Adsorbent}\;\mathrm{Bed}}$$

Breakthrough was quantified by BV_10_, BV_50_, and BV_MCL_ values. BV_10_ is the number of BVs treated until the RSSCT effluent concentration (C) was equivalent to 10% of the RSSCT influent concentration (C_0_). Similarly, BV_50_ is the number of BVs treated until C/C_0_ = 0.5. BV_MCL_ is the number of BVs treated until C first surpassed one of the EPA MCL criteria. BV_10_, BV_50_, and BV_MCL_ were calculated by linear interpolation between sampling points. Media usage rates (MURs; pounds per million gallons (lb/MG)) were estimated based on the mass of media required to treat one million gallons of water if media replacement occurred at BV_MCL_.

## 3 Results and Discussion

### 3.1 PFAS Rejection from Wastewater Effluent by Nanofiltration

The pilot NF system was operated in the flow-through configuration for >45 days, treating wastewater effluent that was periodically spiked with PFAS to evaluate PFAS rejection during high recovery batch experiments. PFAS effective and single pass rejection values vs recovery for the day 30 batch experiments are shown in Figure 2a and b, respectively, and overall batch (90% recovery) rejection values observed on days 1, 15, and 30 are shown in Figure 3. Feed and permeate PFAS concentrations for days 1, 15, and 30 are provided in Table S8 in the SI, and inorganic ion effective rejection values for the day 30 batch experiments are provided in Figure S1 in the SI.

**Figure 2:**
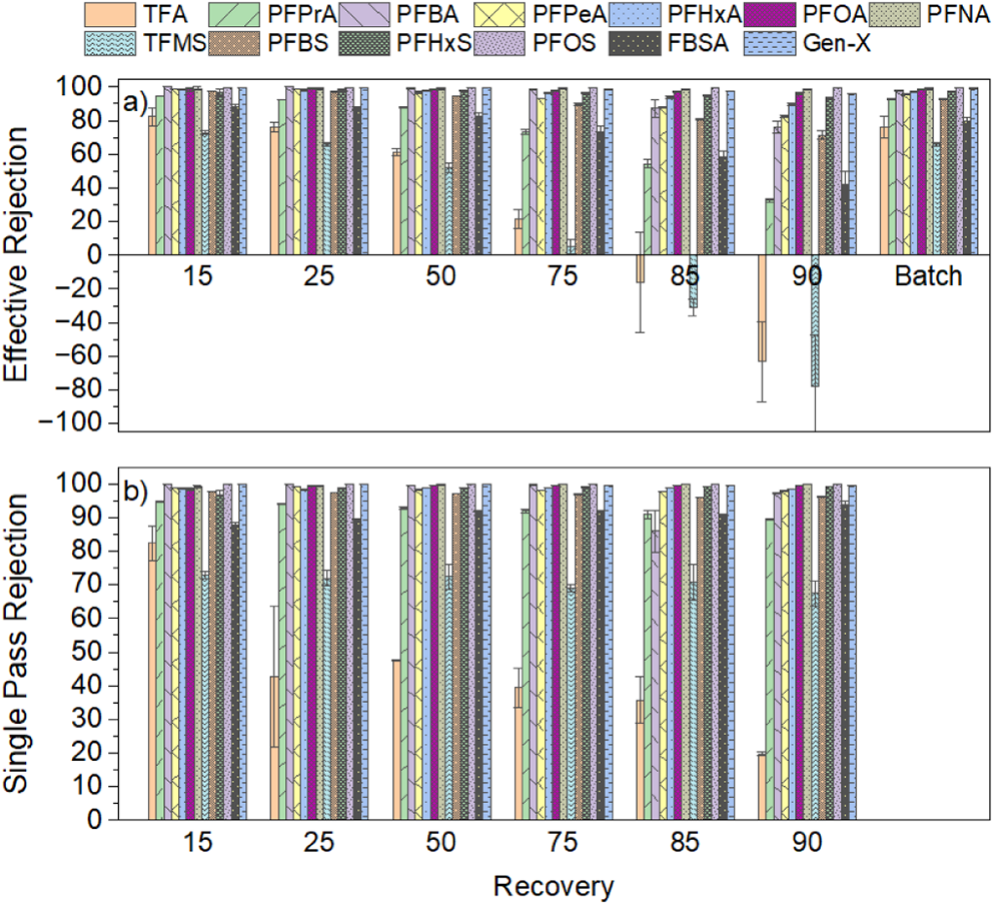
Effect of increasing water recovery on (a) effective rejection of PFAS (eq 2) and (b) single pass rejection of PFAS (eq 1) measured in batch experiments conducted on day 30 of continuous membrane operation. Conditions: 25 LMH, 15% single pass recovery, 2540 NF270 membrane module, and PFAS feedwater concentrations listed in Table 1. Error bars represent the range of values observed in duplicate experiments.

**Figure 3:**
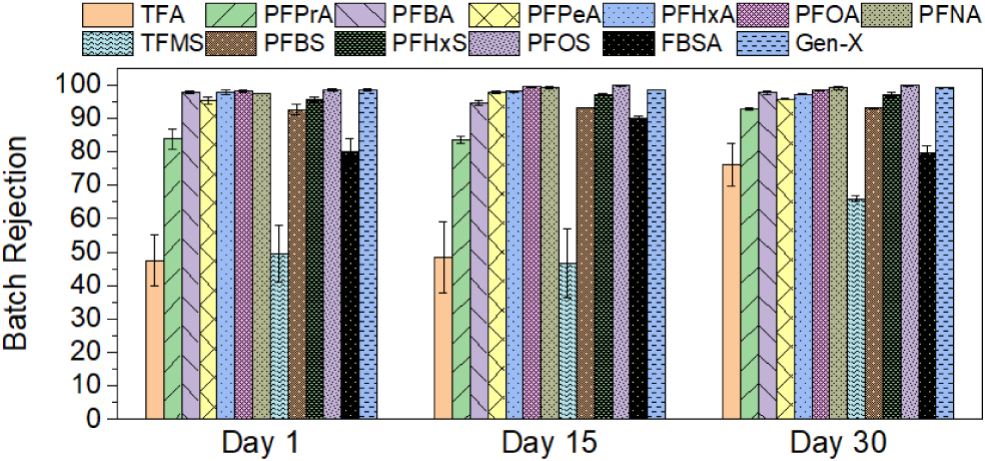
Overall batch PFAS rejection measured in batch experiments conducted on days 1, 15, and 30 of continuous NF system operation. Conditions: 25 LMH, 15% single pass recovery, 2540 NF270 membrane module, and PFAS feedwater concentrations listed in Table 1. Error bars represent the range of values observed in duplicate experiments.

Batch, effective, and single pass rejection values for individual PFAS all increased with the PFAS chain length. Overall batch rejection values on day 30 (Figure 3) exceeded 92% for all PFAS evaluated, except for the shortest chain PFAAs (TFA and TFMS) and the perfluoroalkyl sulfonamide precursor FBSA, which had batch rejection values of 76.2 ± 6.4, 65.9 ± 1.0, and 79.7 ± 2.1%, respectively. Chain length-dependent PFAS rejection has been widely reported, particularly for looser NF membranes like the NF270, with limited data available for ultrashort chain PFAS.^9,11,15^ The lower observed batch rejection of ultrashort TFA and TFMS compared to longer chain PFAS is likely due to the decreased steric exclusion of these low MW structures. However, given that MWs of these ultrashort PFAS are similar to or lower than the reported MWCO of the NF270 membrane (136–340 g/mol^26–30^ ), rejection was higher than expected based on size exclusion alone. TFA and TFMS are anionic at neutral pH (reported p*K*_a_ values <1 ^31^) and electrostatic exclusion from the negatively charged membrane polyamide active layer enhances rejection of these PFAS;^32^ this repulsion also contributes to the high rejection of Gen-X and the other PFAAs evaluated. FBSA rejection values were lower than those observed for PFBA and PFBS, which are similarly sized, because of FBSA’s higher p*K*_a_ (p*K*_a_ = 6.5 ^33^). Thus, at neutral pH, a portion of FBSA exists as the protonated nonionic species, decreasing repulsive electrostatic interactions with the membrane.^20^ Lower rejection of perfluoroalkyl sulfonamides compared to PFAAs has been previously reported,^20^ consistent with the lower rejection of nonionic organic compounds by NF and RO membranes^34,35^ and highlighting the importance of electrostatic interactions for PFAS rejection.

Effective rejection (Figure 2a) decreased at higher water recovery, particularly for short and ultrashort chain PFAS, because of the increased concentration of PFAS in the system during the recirculation of the membrane concentrate. Ultrashort chain TFA and TFMS exhibited negative effective rejection at 90% recovery, meaning instantaneous concentrations of TFA and TFMS in the NF permeate produced at 90% recovery exceeded the concentrations of TFA and TFMS in the spiked feedwater prior to recirculation (see eq 2). Single pass rejection values (Figure 2b), which account for the increasing concentrations of PFAS on the feed side of the membrane during batch recirculation and are more indicative of membrane performance (eq 1), also decreased somewhat with increasing recovery for ultrashort chain compounds but slightly increased with recovery for long chain compounds.

Batch rejection values for background matrix constituents on day 30 varied (Figure S1 in the SI), with high to moderate values for multivalent ions (98.8 ± 0.2% for SO_4_^2–^, 72.9 ± 0.1% for Mg^2+^) and low values for monovalent ions (−1.4 ± 0.3% for Cl^–^; 27.4 ± 0.7% for Na^+^).

Safulko and coworkers evaluated PFAA rejection from tap water spiked with AFFF using the NF270 membrane, observing a decrease in effective PFAS rejection at high water recoveries, particularly for short chain compounds.^15^ However, similar to the present study, short chain PFAS (PFBS) single pass rejection decreased with increasing water recovery, while long chain PFAS (PFOS) single pass rejection increased. The high concentrations of dissolved ions present on the feed side of the membrane at high recovery can decrease electrostatic repulsion by the membrane, which is particularly important for the rejection of short and ultrashort chain PFAS. Moreover, steric exclusion, which is the dominant mechanism for rejection of long chain PFAS, can be enhanced by the complexation of PFAS with divalent cations and DOC^16,17^ or by the reduction of NF effective pore size at high ionic strength.^16^ However, decreased rejection of both PFBS and PFOS has been observed in the presence of divalent anions due to charge shielding and Donnan-enhanced PFAS transport;^15^ therefore, the impact of the water matrix, including specific ions, on PFAS rejection warrants further research.

During the 45 days of NF system operation, membrane permeability declined, as evidenced by an increase in feed pressure and a decrease in temperature-corrected specific flux (TCSF) (Figure 4), indicating that either inorganic scaling, biofouling, or EfOM fouling occurred. However, batch rejection values for PFAS remained consistent between experiments on days 1, 15, and 30 (Figure 3), indicating that fouling or scaling did not have a significant impact on PFAS rejection. Effective and single pass rejection values for the day 1 and day 15 experiments at each recovery set point (Figures S2 and S3 in SI) were also similar to those shown in Figure 2 on day 30. After 40 days of NF operation, membrane cleaning at pH 12 using NaOH and pH 4 using HCl was performed. Cleaning resulted in partial restoration of membrane permeability in terms of feed pressure and TCSF, indicating that a portion of the scaling or fouling layer was removed. Further optimization of membrane cleaning, and antiscalant (reduction of scalant formation) and/or chloramine (reduction of biofouling) use would be required to maintain consistent membrane permeability during long-term operation.^36–38^

**Figure 4:**
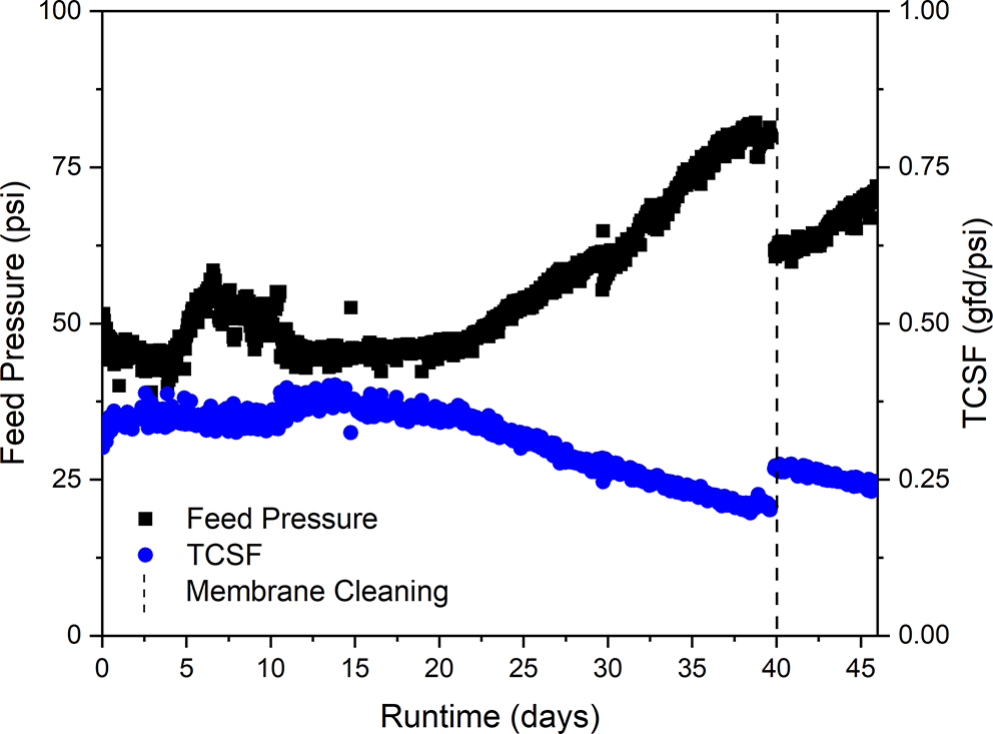
NF pilot system feed pressure (left axis) and temperature-corrected specific flux (TCSF; right axis) over the 45 day duration of continuously treating wastewater effluent. The vertical dashed line indicates when sequential NaOH (pH 12) and HCl (pH 4) cleaning was performed. Conditions: 25 LMH, 15% single pass recovery, and 2540 NF270 membrane module.

Several groups have investigated the effect of model biofoulants on PFAS rejection in bench scale experiments.^16,39^ Bovine serum albumin (BSA) and sodium alginate (SA) were found to improve PFOS rejection by increasing steric exclusion after the formation of a fouling layer on the membrane surface, whereas PFBS rejection increased with the addition of SA but decreased in the presence of BSA due to differences in the surface charge of the fouled membranes.^16^ In a separate study, PFOS rejection increased with humic acid (HA) and BSA addition, while PFBS rejection increased with HA but decreased with SA addition.^39^ These results indicate that foulants may improve rejection by enhancing steric exclusion of long chain PFAS through coordination of PFAS with natural organic matter (NOM) or by NOM reducing the membrane’s effective pore size, but have varied impacts on electrostatic repulsion, which is important for the rejection of short chain PFAS like PFBS. Studies evaluating PFAS rejection in real wastewater matrices by spiral wound membranes are less common. PFAS rejection by the Filmtec NF90 membrane was observed to be higher in semiconductor manufacturing wastewater compared to deionized water (5 mM NaCl, 1 mM NaHCO_3_).^9^ This was attributed to increased coordination of PFAS with EfOM or inorganic ions increasing steric exclusion, but the limited volume of water treated precluded analysis of the long-term impact of membrane fouling on PFAS rejection and electrostatic interactions are less important for PFAS rejection by the tighter NF90 compared to the NF270 membrane used in the present study.^9^ Liu et al. found that PFAS rejection by the NF270 membrane did not change significantly over 30 days of continuous treatment of contaminated groundwater, but organic fouling or biofouling was not observed over the duration of testing.^11^ The stability of batch rejection values in the current study suggests that PFAS rejection was not significantly impacted by the fouling or scaling that occurred; however, the membrane was not continuously exposed to PFAS for the duration of the pilot, so the impact of PFAS adsorption on rejection was not determined. Additional research evaluating PFAS rejection in a continuous PFAS-impacted wastewater stream is recommended.

While the NF system provided high rejection of PFAS, particularly long chain compounds that are subject to regulation, concentrations in the combined permeate (PFOA = 16.1 ± 0.1 ng/L, PFOS = 10.7 ± 0.0 ng/L, PFHxS = 29.3 ± 0.1 ng/L, PFNA = 10.4 ± 0.9 ng/L, and Gen-X = 21.4 ± 0.3 ng/L) from batch NF experiments still exceeded EPA MCLs due to the high PFAS concentrations in the feedwater. Therefore, additional treatment would be required if the NF permeate is intended for use in applications such as augmentation of drinking water supplies.

### 3.2 PFAS Removal from Wastewater Effluent by Adsorbents Alone

Direct PFAS removal from the wastewater effluent using GAC and IX (without NF treatment first) is shown in Figures 5a–c and 6a–c, respectively. For each plot, data for PFCAs are shown in panel a; PFSAs are shown in panel b; and FBSA, Gen-X, and the calculated hazard index of the PFAS mixture are shown in panel c. Absolute PFAS concentrations in the RSSCT effluents are shown instead of normalized C/C_*o*_ values to facilitate comparison with the combined treatment train (to be discussed later) for meeting the various PFAS MCLs. Corresponding breakthrough curves represented as C/C_*o*_ values are provided in Figure S4 in the SI. Near immediate breakthrough of all PFAS was observed for GAC as quantified by the estimated BV_10_ and BV_50_ values (Table 2 and Figure S5). For GAC, BV_10_ values for TFA, PFPrA, and TFMS were 25 ± 9, 67 ± 26, and 72 ± 19BVs, respectively, and BV_10_ values increased with increasing chain length for PFCAs and PFSAs up to 283 ± 73 and 467 ± 43 BVs for PFNA (longest chain PFCA studied) and PFOS (longest chain PFSA), respectively. Treatment with IX was better than GAC, but breakthrough still occurred fairly quickly. IX BV_10_ values for TFA, PFPrA, and TFMS were 130, 129, and 108 BVs, respectively, and BV_10_ values increased with increasing chain length up to 34,800 and 89,600 for PFNA and PFOS, respectively. In addition to the trend of increasing BV_10_ values with increasing chain length, BV_10_ values were higher for PFSAs than for the equivalent length PFCAs in certain cases. FBSA and Gen-X also broke through quickly, with GAC BV_10_ values of 299 ± 41and 178 ± 18, respectively, and IX BV_10_ values of 14,400 and 15,400, respectively. It can be concluded from these findings that direct treatment of wastewater effluent using GAC or IX adsorbents alone is not practical due to the rapid breakthrough and correspondingly high media usage rates.

**Table 2:** BV_10_ and BV_50_ Values for GAC And IX for RSSCTs Conducted for Direct Treatment of Wastewater Effluent Spiked with PFAS and BV_10_ Values for NF-Adsorbent Treatment Train and PFAS-Spiked NF Permeate

	GAC (F400)				IX (CalRes 2301)			
	Direct treatment of PFAS-spiked wastewater effluent		Treatment train	PFAS-spiked permeate	Direct treatment of PFAS-spiked wastewater effluent^a^		Treatment train	PFAS-spiked permeate
	BV_10_	BV_50_	BV_10_	BV_10_	BV_10_	BV_50_	BV_10_	BV_10_
TFA	25 ± 9	123 ± 9	102 ± 11	123 ± 12	130	669	2,130 ± 160	1,190 ± 30
PFPrA	67 ± 26	420 ± 49	215 ± 65	287 ± 63	129	644	8,080^b^	5,160 ± 90
PFBA	181 ± 34	791 ± 283	820 ± 475	3,130 ± 560	4,150	8,380	19,700 ± 3,300	13,000 ± 1,000
PFPeA	196 ± 20	1,110 ± 80	2,220 ± 2,220	16,900 ± 4,100	10,300	19,300	81,800 ± 5,900	51,900 ± 3,400
PFHxA	284 ± 15	2,450 ± 330	1,890 ± 680	>23,400	10,600	35,100	35,500 ± 9,300	150,000 ± 10,000
PFOA	246 ± 34	1,760 ± 220	9,600 ± 1,500	>23,400	29,800	66,600	>450,000	170,000 ± 6,000
PFNA	283 ± 73	1,890 ± 900	9,360 ± 1,270	>23,400	34,800	87,800	>450,000	165,000 ± 9,000
TFMS	72 ± 19	396 ± 17	272 ± 212	304 ± 82	108	602	144,000 ± 11,000	93,700 ± 5,900
PFBS	273 ± 32	1,790 ± 100	5,070 ± 3,590	>23,400	52,300	110,000	>450,000	200,000 ± 7,000
PFHxS	305 ± 37	1,830 ± 60	10,200 ± 7,600	>23,400	72,200	205,000	>450,000	203,000 ± 7,000
PFOS	467 ± 43	4,620 ± 850	>35,100	>23,400	89,600	352,000	>450,000	223,000 ± 4,000
FBSA	299 ± 41	2,080 ± 250	3,820 ± 2,910	>23,400	14,400	27,800	169,000 ± 26,000	115,000 ± 3,000
Gen-X	178 ± 18	813 ± 103	2,920 ± 1,870	21,300 ± 4,000	15,400	29,500	334,000 ± 30,000	140,000 ± 6,000

a Uncertainty not available for IX direct treatment of wastewater effluent due to the mechanical failure of a duplicate experiment.

b Uncertainty not available due to an analytical error for the duplicate sample.

**Figure 5:**
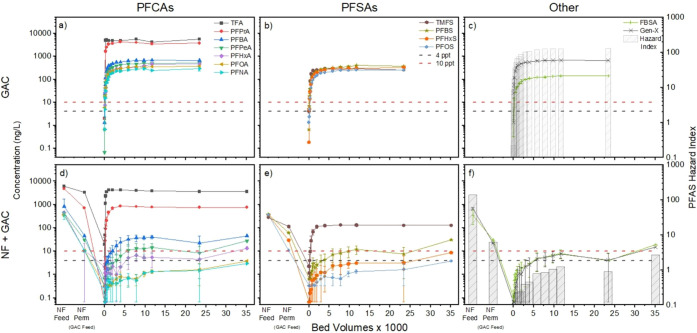
RSSCT breakthrough curves for (a–c) direct GAC treatment of wastewater effluent spiked with PFAS versus (d–f) combined NF-GAC treatment train; the left side of panels (d–f) shows the decrease in the concentration of each PFAS during NF treatment before introducing the permeate as feedwater for the RSSCT. NF conditions are described in Figures 2–3. GAC RSSCT conditions: 20 min simulated EBCT, 4 gpm/ft^2^HLR. Error bars represent values observed in duplicate experiments. Corresponding breakthrough data plotted in terms of C/C_0_ are provided in Figures S4 and S6 in SI.

**Figure 6:**
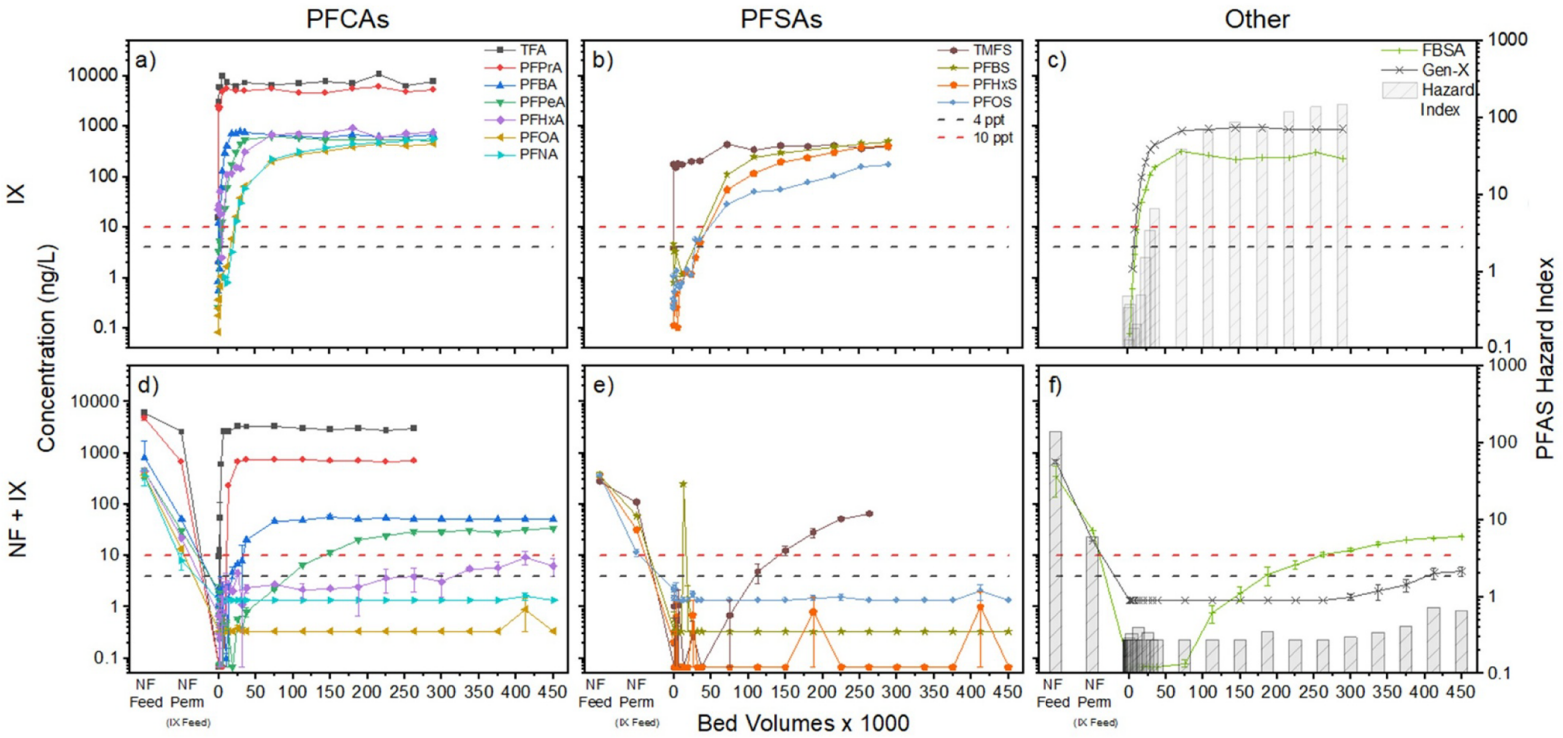
RSSCT breakthrough curves for (a–c) direct IX treatment of wastewater effluent spiked with PFAS versus (d–f) combined NF-IX treatment train; the left side of panels (d–f) shows the decrease in the concentration of each PFAS during NF treatment before introducing the permeate as feedwater for the RSSCT. NF conditions are described in Figures 2–3. IX RSSCT conditions: 2 min simulated EBCT, 9.25 gpm/ft^2^ HLR. Error bars represent the range of values observed in duplicate experiments. Constant low concentrations for PFNA, PFOA, PFOS, PFHxS, PFBS, and Gen-X in parts (d–f) are due to analytical detection limits. Corresponding breakthrough data plotted in terms of C/C_0_ are provided in Figures S4 and S6 in SI.

The BV_10_ values observed here when treating PFAS-spiked wastewater effluent are lower than reported literature values for the treatment of PFAS-contaminated groundwater with the same adsorbents, particularly for GAC. A previous study comparing PFAS removal from groundwater by F400 GAC and Calres2301 IX reported PFOS BV_10_ values >25,000 (>50 times the PFOS BV_10_ value for direct treatment of wastewater in this study) and >100,000, respectively.^40^ Although breakthrough was accelerated in PFAS-spiked wastewater, the higher BVs of water treated before breakthrough with increasing chain length within the respective PFAS class and for PFSAs compared to PFCAs of similar chain length are consistent with previous studies.^12–14^ Improved adsorption of longer chain compared to shorter chain PFAS can be attributed to the increased hydrophobicity of long chain PFAS. This chain length dependence was observed for the adsorption of ultrashort, short, and long chain PFAS by GAC and IX in this study. For IX, higher adsorption of PFSAs compared to PFCAs was most evident for short and long chain PFAS, while ultrashort PFAS experienced similarly fast breakthrough irrespective of the functional group. For GAC, differences in adsorption between PFAS classes were more muted, but PFSA BV_10_ values still exceeded values for PFCAs of equivalent fluorinated carbon chain length (Figure S5). Improved adsorption of PFSAs compared to PFCAs is due to the improved electrostatic interactions of the sulfonate headgroup;^14^ therefore, the higher adsorption of PFSAs observed for IX demonstrates the importance of electrostatic interactions between PFAS and IX. The overall fast breakthrough of PFAS for both GAC and IX can be attributed to the comparatively high concentrations of EfOM and inorganic ions present in the wastewater effluent (Table 1). Accelerated breakthrough of PFAS in water matrices with elevated DOC and inorganic ions, such as the wastewater effluent evaluated in these experiments, compared to less solute-rich water matrices, has previously been reported.^6,10,12–14^ DOC can compete for or block sorption sites in GAC and IX, while inorganic anions can disrupt electrostatic interactions between PFAS and IX. Additional discussion of the impact of the water matrix on adsorption mechanisms is provided in later sections.

### 3.3 PFAS Removal from Wastewater Effluent by Sequential Nanofiltration and Adsorbents

Results presented above demonstrate that NF filtration alone or treatment with adsorbents alone may provide insufficient removal or require frequent media replacement to meet strict PFAS limits when addressing solute-rich water matrices, such as wastewater effluent with elevated PFAS concentrations. In contrast, combining NF sequentially with adsorbents can provide more effective PFAS removal and extend the adsorbent lifetime when treating these complex water matrices. Here, the combined NF-adsorbent treatment trains were evaluated by conducting RSSCTs with GAC and IX on permeate from the NF batch experiments described in Section 3.1. The results from these experiments are shown in Figures 5d–f and 6d–f, where concentrations of individual analytes are tracked across the full treatment train, including before and after NF membrane treatment (far left side of each panel) and then in the effluent of the RSSCTs. For each of these figures, data for PFCAs are shown in panel d; PFSAs are shown in panel e; and FBSA, Gen-X, and the calculated hazard index are shown in panel f. By showing absolute PFAS concentrations in the RSSCT effluents instead of normalized C/C_*o*_ values, results highlight the performance of the combined treatment train for meeting the various PFAS MCLs and facilitate comparison of treatment train data (lower panels of each figure) with treatment by adsorbents alone (upper panels). The flat or nearly flat breakthrough curves observed for PFOA, PFNA, PFBS, PFHxS, PFOS, and Gen-X in Figure 6d–f are due to measured concentrations being at or near the analytical detection limit. Corresponding breakthrough curves with C/C_*o*_ values are available in Figure S6 in the SI.

For PFAS with regulatory limits, PFOS concentrations decreased from 359 ± 16 ng/L in the NF feed to 10.7 ± 0.0 ng/L in the NF permeate, which served as the feed for the GAC and IX RSSCTs. GAC RSSCT effluent PFOS concentrations (Figure 5e) remained below the 4 ng/L PFOS MCL for the duration of the experiments (35,100 BVs). Similarly, PFOA decreased from 364 ± 35 ng/L in the NF feed to 10.7 ± 0.0 ng/L in the permeate and remained below 4 ng/L in the GAC RSSCT effluent (Figure 5d). The hazard index (Figure 5f) decreased from 137 in the NF feed to 6.1 in the permeate and then remained below 1.0 in the GAC RSSCT effluent until 9,300 BVs; exceedance of the hazard index was primarily due to Gen-X (6.2 ± 3.3 ng/L). For IX (Figure 6d–f), PFOS and PFOA concentrations remained below 4 ng/L and the hazard index remained below 1.0 in the RSSCT effluent for the duration of the experiments (450,000 BVs). This translates to a media usage rate of 484 lb/MG of water treated for GAC and <12.4 lb/MG for IX (final sample point BVs was used) if media changeout occurred the first time any MCL was violated.

TFA, PFPrA, and TFMS concentrations in the GAC RSSCT effluent increased faster than the concentrations of longer chain compounds, reaching 4,180 ± 210, 697 ± 94, and 113 ± 19 ng/L, respectively, at 1,950 BVs, before plateauing for the duration of the experiment. TFA and PFPrA reached concentrations of 3,300 ± 320 and 686 ± 49 ng/L, respectively, in the IX RSSCT effluent at 25,000 BVs before plateauing; however, TFMS remained below 70 ng/L for the duration of the experiment. High TFA and PFPrA concentrations in the GAC and IX RSSCT effluent can be explained by the higher concentrations spiked into the NF feed and the lower rejection of these compounds by the NF270 membrane, decreasing from 6,080 ± 340 to 3,300 ± 50 ng/L for TFA and 4,810 ± 430 to 701 ± 18 ng/L for PFPrA from NF feed to permeate. TFMS was spiked into the NF feed at a lower concentration but was also poorly rejected by the membrane, decreasing from 283 ± 21 to 113 ± 6 ng/L from the NF feed to permeate. Moreover, ultrashort chain compounds were poorly retained by the adsorbents, leading to the rapid breakthrough of TFA, PFPrA, and TFMS in GAC RSSCTs, and TFA and PFPrA in IX RSSCTs. IX retained TFMS to a similar degree as the longer chain PFBA due to the improved interaction of the sulfonate headgroup with the IX resin.^14^

Combining NF with GAC or IX treatment of NF permeate provided a robust multi barrier process that produced low PFAS concentrations in the final effluent. NF combined with GAC resulted in 99.1, 99.0, 94.4, and 91.7% removal of PFOA, PFOS, PFBA, and PFBS up to 35,100 BVs, respectively, while NF combined with IX resulted in >99.9, >99.6, 93.6, and >99.9% removal of PFOA, PFOS, PFBA, and PFBS up to 450,000 BVs, respectively, when comparing NF feed concentrations to concentrations measured during the final sampling event for each RSSCT. Membranes and adsorbents have been previously combined for PFAS treatment, but with GAC or IX treatment of membrane concentrate, not permeate.^41^ These authors reported average rejection exceeding 99% of C4–C8 PFAAs by the NF270 in contaminated groundwater at 78% recovery; however, rejection values for individual PFAS were not specified and PFCA concentrations were often at or near detection limits in the NF feed. A follow-up study found similar results using both NF270 and NF90 membranes combined with GAC and IX treatment of NF concentrate.^42^ Verliefde and coworkers evaluated GAC treatment of pharmaceutical micropollutants in NF permeate, finding that anionic structures were better rejected than neutral and positively charged structures,^43^ similar to the trends in PFAS rejection vs p*K*_a_ observed in the present work. Lower rejection in high recovery (80%) experiments was attributed to higher concentrations of dissolved ions in the feed and deposition of NOM on the membrane surface shielding membrane charge. Near complete removal (>98%) of pharmaceuticals present in the NF permeate by GAC was attributed to the removal of high MW NOM by the initial NF step, but the GAC pilot was not operated long enough (4 days) to evaluate breakthrough.^43^ Tajdini and coworkers evaluated ozone (O_3_) and biologically activated carbon filtration (BAF) as pretreatment for PFAS removal by GAC and IX in wastewater effluent from the same source evaluated in this work, finding lower improvements in adsorption compared to direct adsorptive treatment of the wastewater effluent (5 to 5.3x for GAC and 1.6 to 4x for IX for PFAAs ranging C4–C8) than the improvements resulting from NF pretreatment in the present study (see Section 3.4). Improved PFAS adsorption was attributed to the transformation of hydrophobic EfOM to hydrophilic EfOM by O_3_ and biotransformation of EfOM by BAF, resulting in decreased blocking of adsorption sites and decreased competition between EfOM and PFAS for adsorption.^14^

### 3.4 PFAS Adsorbent Performance Improvement with NF Pretreatment of Nontarget Wastewater Constituents

Because of the low PFAS concentrations remaining in NF permeate during the treatment of PFAS-spiked wastewater effluent, it was unclear if the observed improvements in adsorbent performance were principally due to reductions in PFAS concentrations or reductions in nontarget water constituents in the permeate feedwater. To address this, a third set of RSSCTs was performed wherein NF permeate was collected during normal system operation and spiked with PFAS (rather than spiking wastewater before membrane filtration) to isolate the effect of the reduction of EfOM and other nontarget solutes by NF on adsorbent performance while maintaining elevated PFAS concentrations. Breakthrough curves from these RSSCTs (Figure S7 in SI) were used to calculate BV_10_ values for individual PFAS (Table 2) and performance improvement factors (Figure 7) relative to BV_10_ values observed for the direct treatment of PFAS-spiked wastewater effluent:

6 $$\mathrm{Improvement}\;\mathrm{Factor}=\frac{BV_{10}\;\mathrm{RSSCT}\;\mathrm{for}\;\mathrm{spiked}\;\mathrm{NF}\;\mathrm{permeate}}{BV_{10}\;\mathrm{RSSCT}\;\mathrm{for}\;\mathrm{WW}\;\mathrm{effluent}}$$

**Figure 7:**
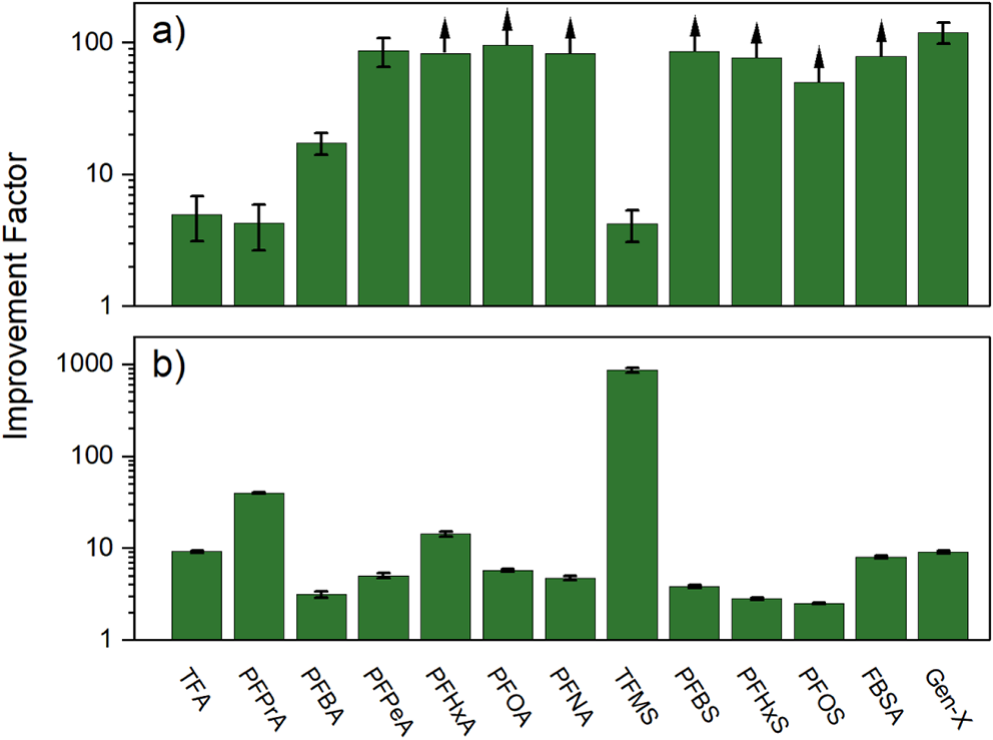
Performance improvement factors (eq 6) calculated from the increase in BV_10_ values for (a) GAC and (b) IX RSSCTs on NF permeate compared to direct treatment of wastewater effluent. Error bars represent the range of values observed in duplicate experiments. Upward arrows indicate lower limit estimates for the performance improvement factors since the breakthrough of that specific PFAS was not reached.

Upward arrows in Figure 7a indicate lower limit estimates for the performance improvement factors since 10% breakthrough of that specific PFAS was not reached in the GAC RSSCT experiment before the final sampling point (23,400 BVs). The use of NF as pretreatment for GAC resulted in much larger improvement factors for long chain compared to short chain PFAAs. While moderate improvement factors were observed for TFA (5.0 ± 1.9), PFPrA (4.3 ± 1.6), and TFMS (4.2 ± 1.1), much larger values were observed for PFOA (>95.2), PFNA (>82.8), and PFOS (>50.2). The opposite trend was noted for IX, where the use of NF as pretreatment led to improvement factors of 9.2 ± 0.2, 40.0 ± 0.7, and 865 ± 55 for TFA, PFPrA, and TFMS, respectively, but smaller improvement factors for the longer chain PFOA (5.7 ± 0.2), PFNA (4.7 ± 0.3), and PFOS (2.5 ± 0.0). However, it is worth noting that these lower improvement factors observed for IX compared to GAC are balanced by the fact that the IX resin performed much better for long chain PFAAs without NF pretreatment (Table 2; e.g., IX BV_10_ = 34,800 for PFNA).

PFAS adsorption by GAC improved in the NF permeate compared to direct treatment of wastewater effluent, presumably due to the low EfOM concentration in the NF permeate (DOC = 0.9 mg/L) compared to wastewater effluent (DOC = 4.2 mg/L). DOC can block pores within the GAC microstructure, rendering them unavailable for PFAS adsorption. Moreover, DOC may directly compete with PFAS for GAC adsorption sites.^13,14^ The removal of EfOM by NF pretreatment likely decreased pore blocking and competitive adsorption, resulting in higher BVs until breakthrough in membrane permeate compared to wastewater effluent. Higher BVs before PFAS breakthrough have previously been observed for GAC treating groundwater compared to surface water and wastewater with higher DOC, consistent with the results of this study.^6,12^ Adsorption of PFSAs and long chain PFCAs increased more than short chain PFCAs in groundwater compared to surface water, which was attributed to direct competition between NOM and highly adsorbed PFAS (PFSAs and long chain PFCAs) for sorption sites with lower adsorption potential (e.g., adsorption sites in large pores).^12^ This same mechanism could also provide an explanation for the greater GAC improvement factors observed for longer chain PFAS in this study as a result of NF pretreatment. NF is effective for removal of high MW EfOM,^43^ which may directly compete with highly adsorbed PFAS, resulting in the significant improvements in adsorption observed in spiked NF permeate.

Greater improvement factors for IX were observed for ultrashort chain compared to longer chain PFAS. Because IX adsorption is governed by both hydrophobic and electrostatic interactions,^10,44^ decreased EfOM and inorganic ion (particularly multivalent anions, as shown in Table S9 in SI) concentrations in NF permeate resulted in better adsorption, as has been observed in previous studies. For example, Cheng et al. evaluated the impact of water chemistry on the adsorption of PFAS by IX and observed faster PFAS breakthrough in matrices with elevated dissolved organic matter (DOM) and nitrate. Adsorption of short chain PFAS was more negatively impacted by nitrate addition, highlighting the importance of electrostatic interactions, while longer chain PFAS was more negatively impacted by DOM, which can hinder hydrophobic interactions.^10^ The high removal of charge dense multivalent anions that could outcompete PFAS for anion exchange sites (e.g., 98.8% rejection of SO_4_^2–^) by NF pretreatment could explain the significant improvements in the adsorption of ultrashort PFAS by IX observed in this study. Murray et al. found a negative correlation between adsorbent performance and DOC and anion concentration when evaluating GAC and IX for the removal of PFAS from two groundwaters, wastewater effluent, membrane concentrate, and landfill leachate, and that GAC was more negatively impacted than IX.^13^ Their analysis included only short and long chain PFAS (not ultrashort); therefore, their findings match the higher improvement factors observed for GAC compared to IX in this work. While the calculated improvement factors confirm that NF improves GAC and IX performance by removing EfOM and other inorganic ions, it is difficult to attribute the improvement to specific matrix components due to the complex nature of the wastewater.

### 3.5 Technology Implications

Municipal and industrial wastewaters are important vectors for PFAS release into the environment. However, PFAS treatment in wastewater (treated or untreated) is more challenging compared with less solute-rich matrices (e.g., groundwater) due to elevated concentrations of EfOM and inorganic ions, potentially requiring the combination of multiple treatment barriers to meet strict discharge limits. This work demonstrated that an integrated treatment train consisting of NF followed by GAC or IX polishing treatment of NF permeate effectively removes PFAS from wastewater effluent, producing effluent that meets strict regulatory limits being implemented for drinking water. IX provided superior removal of anionic PFAS compared to GAC from NF permeate, but additional factors should be considered when evaluating adsorbents. A variety of zwitterionic and cationic PFAS exist that may not be effectively removed by anion exchange processes.^45^ Moreover, adsorption of other organic co-contaminants present in wastewater matrices should be considered.^46^ Finally, cost analysis should be performed prior to choosing an adsorbent due to the differences in unit prices of GAC, IX, and other media.^13^ NF pretreatment decreased media usage rates, but media regeneration (e.g., thermal regeneration of GAC or solvent regeneration of IX) or disposal through incineration or landfilling at a hazardous waste facility would likely still be required for full scale operation. The PFAS laden concentrate produced from the high recovery NF process is another waste stream that may require destructive treatment. Evaluation of a continuous source of PFAS impacted wastewater effluent is recommended to confirm the performance trends observed in this work. Moreover, future research should include analysis of priority co-contaminants that may exist in PFAS-contaminated municipal or industrial wastewater streams (i.e., pharmaceuticals found in municipal wastewater or onium photo acid generating compounds found in semiconductor manufacturing wastewater), advance efforts to identify specific non-target constituents that most impact PFAS adsorption through variation of individual matrix components,^10^ and determine the ability of pretreatment to remove these constituents.

## Supplementary Material



## Notes

Additional details on materials and methods and additional NF and RSSCT experimental data (PDF)

## ◆

Rice University, Department of Civil and Environmental Engineering, Houston, TX, 77005, USA
