# Supplementary material for: Combining Nanofiltration and Adsorbents to Achieve Effective PFAS Removal in Wastewater Effluent
Source: Environ Sci Technol. 2026 Apr 10;60(16):12587–97. doi: 10.1021/acs.est.5c17011 (PMC13130956; doi:10.1021/acs.est.5c17011)
Supplement: Supplementary file 1 [file es5c17011_si_001.pdf]

Supporting Information

Combining nanofiltration and adsorbents to achieve effective PFAS removal in wastewater effluent

Aron M. Griffin[1,2]*, Jaidyn Elbers[1], Christopher Bellona[1], Timothy J. Strathmann[1]

[1]Colorado School of Mines, Department of Civil and Environmental Engineering, Golden, CO, 80401, USA.

[2]Current address: Rice University, Department of Civil and Environmental Engineering, Houston, TX, 77005, USA.

April 2026

9 Tables, 7 Figures, 11 Pages

*Corresponding author. Email: aron.griffin@rice.edu; Phone: (713)-348-4949

Section S1: Properties of experimental membrane and adsorbents.

Table S1: Properties of the Filmtec NF270 membranes.

Parameter	Value
Isoelectric Point	3-4[1,2]
Membrane Material	Polyamide Thin Film Composite
pH Range*	3-10
$MgSO_4$ Rejection*	97%
NaCl Rejection *	30.0-58.0%
Pore Size (nm)	0.36-0.44[3,4]
MWCO (Da)	136-340[3–7]

*Provided by manufacturer

Table S2: Properties of Calgon F400 GAC.

Parameter	Unit	Value
BET surface area	m^2/g	785[8]
Total pore volume	cm^3/g	0.27[8]
Micropore volume	cm^3/g	0.05[8]
Mesopore volume	cm^3/g	0.05[8]
Macropore volume	cm^3/g	10[8]
Bulk density*	g/mL	0.54
Iodine number*	mg/g	≥1000

*Provided by manufacturer

Table S3: Properties of Calres 2300 IX provided by manufacturer.

Parameter	Unit	Value
Type of resin	-	macroporous strong base anion
Ionic form	-	chloride
Base structure polymer	-	styrene
Functional group	-	tributylamine
Regenerability	-	single use
Particle size	μm	580 ± 50
Measured shipping product density	g/L	670
Ion exchange capacity	eq/L	≥0.51
Water retention capacity	wt%	48-60

Section S2: PFAS characteristics

Table S4: Characteristics of PFAS spiked into membrane and adsorbent experiments.

Class	Compound Name	Abbreviation	n	MW g/mol	Structure
PFCAs	Trifluoroacetic acid	TFA	0	113	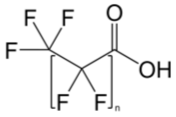
	Perfluoropropanoic acid	PFPrA	1	163	
	Perfluorobutanoic acid	PFBA	2	213	
	Perfluoropentanoic acid	PFPeA	3	263	
	Perfluorohexanoic Acid	PFHxA	4	313	
	Perfluorooctanoic acid	PFOA	6	413	
	Perfluorononanoic acid	PFNA	7	463	
PFSAs	Trifluoromethane sulfonic acid	TFMS	0	149	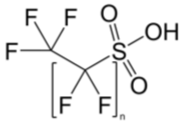
	Perfluorobutane sulfonic acid	PFBS	3	299	
	Perfluorohexane sulfonic acid	PFHxS	5	399	
	Perfluorooctane sulfonic acid	PFOS	7	499	
FOSAs	Perfluorobutane sulfonamide	FBSA	-	298	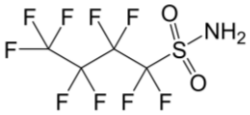
PFECAs	Hexafluoropropylene oxide dimer acid	Gen-X	-	347	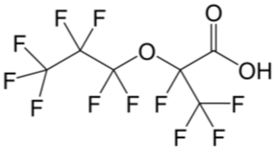

Section S3: Tertiary treated wastewater effluent characterization

Table S5: Tertiary treated wastewater effluent characterization.

Date	sCOD mg/L	sTN mg/L	TP mg/L	NH_4 mg/L	NO_3 mg/L	Alkalinity mg/L	pH
10/25/2024	38.5 ± 0.6	4.89 ± 0.02	27.80 ± 0.10	0.77 ± 0.00	3.41 ± 0.00	45.6 ± 0.8	6.8
11/8/2024	10.9 ± 0.0	11.00 ± 0.00	7.02 ± 0.04	1.67 ± 0.00	8.63 ± 0.04	39.3 ± 0.9	6.7
11/15/2024	16.0 ± 0.2	13.30 ± 0.00	7.13 ± 0.04	1.83 ± 0.01	9.80 ± 0.00	19.6 ± 0.0	6.5
11/22/2024	21.0 ± 0.2	10.90 ± 0.00	16.15 ± 0.01	1.05 ± 0.01	4.73 ± 0.00	69.1 ± 0.4	6.9
11/30/2024	10.5 ± 0.3	5.76 ± 0.03	9.89 ± 0.03	7.38 ± 0.00	4.11 ± 0.01	52.4 ± 0.4	7.0
12/6/2024	24.0 ± 0.3	12.40 ± 0.03	10.09 ± 0.01	7.38 ± 0.01	3.86 ± 0.01	71.7 ± 0.3	7.3

Section S4: Rapid small scale column testing operational details

Table S6: Rapid small scale column testing operational details.

Parameter	Unit	F400	CalRes 2301
US mesh	-	100 x 140	200 x 230
Particle size	μm	57.8	68.3
Column diameter	cm	0.43	0.43
Bed volume	ml	0.147	0.106
Flow rate	ml/min	2.39	5.52
Simulated EBCT	min	20	2
Scaling factor	-	18	10.2
Hydraulic loading rate	gpm/ft^2	4.0	9.25

Section S5: Detailed description of methods

Per- and polyfluoroalkyl substances (PFAS) were analyzed using a SCIEX 5500 QTRAP Triple Quad mass spectrometer. Calibration curves ranging from 0.067 to 6,667 ng/L with mass labeled internal standards provided by 3M, Wellington Laboratories, Fluobon, and Synquest facilitated quantification of PFAS concentrations. LC-MS/MS files were analyzed using SCIEX OS Analyst. Lab blank and double blanks were prepared using the same solvents as the samples to ensure no contamination. Lab control samples were prepared using a known concentration of standards (300 pg in 1.5mL) to track instrument response throughout the run. Internal standards were included in each sample to correct PFAS concentrations.

An established method was used to analyze short and long chain (≥C3) PFAS.[9] This method used a reversed phase Phenomenex C18 column with a 5 μm particle size and 110 Å pore size. A gradient method was used with a total flow of 0.6 mL/min that alternated between majority aqueous and organic elution phases. The aqueous mobile phase consisted of 20 mM ammonium acetate in Optima LC-MS grade water (Fisher Scientific) and the organic phase was Optima LC-MS grade methanol (Fisher Scientific). The C18 column performs well for short and long chain compounds due to their hydrophobic nature.

The ultrashort chain PFAS compounds were measured using a Rspak JJ-50 2D (Shodex) multi-mode analytical column with 5 μm particle size and 100 Å pore size with an aqueous mobile phase consisting of 40 mM ammonium acetate in Optima LC-MS grade water (Fisher Scientific) and an organic phase of Optima LC-MS grade acetonitrile (Fisher Scientific). This followed an isocratic method with a total eluent flow of 0.2 mL/min, 80% organic eluent, and 20% aqueous eluent that was recently developed at Mines.[10]

Dissolved cations were measured using a Dionex ICS90 ion chromatography and cations were measured using a Perkin-Elmer 5300 inductively coupled plasma-atomic emission spectrometer (ICP-AES). Dissolved organic carbon (DOC) was measured using a Shimadzu TOC-L analyzer.

Section S6: Inorganic ion effective rejection data for Day 30 batch experiments.

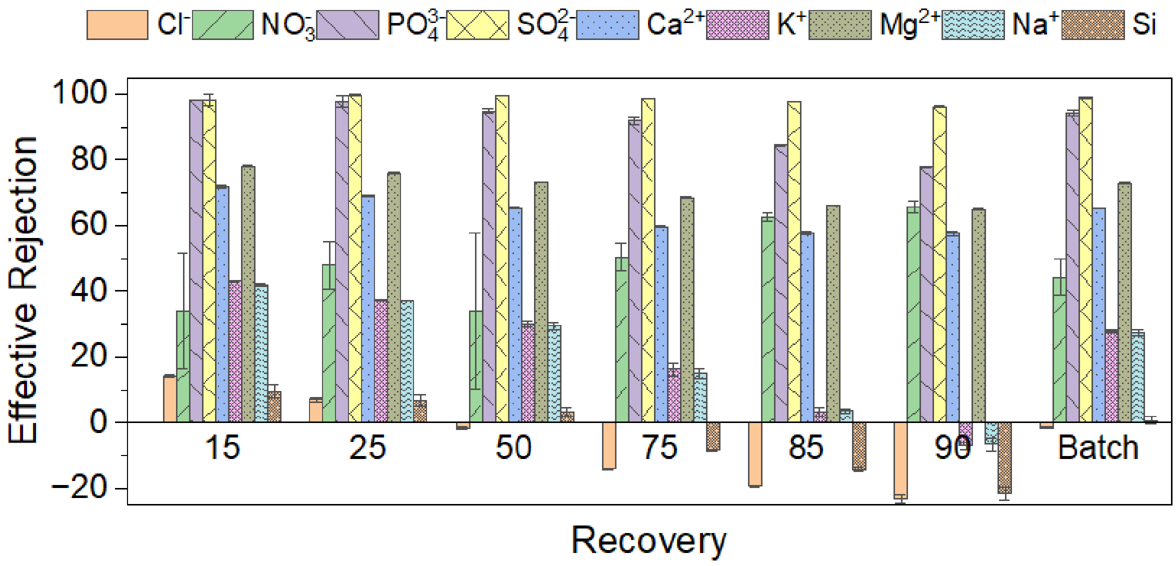

Figure S1: Effect of increasing water recovery on effective rejection (eq 2) of background water constituents measured in batch experiments conducted on day 30 of continuous membrane operation. Conditions: 25 LMH, 15% single pass recovery, 2540 NF270 membrane module. Error bars represent range of values observed in duplicate experiments.

Table S7: Characteristics of wastewater effluent used as feed water for NF batch experiments conducted on day 30.

**Parameter**	**Value** (mg/L)
Cl^-	102 ± 3
NO_3^-	52.8 + 3.0
PO_4^{3-}	16.9 + 0.3
SO_4^{2-}	145 ± 6
Ca^{2+}	25.2 ± 0.3
K^+	8.27 + 0.01
Mg^{2+}	6.05 ± 0.07
Na^+	34.2 ± 0.4
P	2.73 ± 0.10
S	27.3 ± 0.7
Si	1.84 ± 0.02

Section S7: Effective and single pass rejection data for Day 1 and 15 batch experiments.

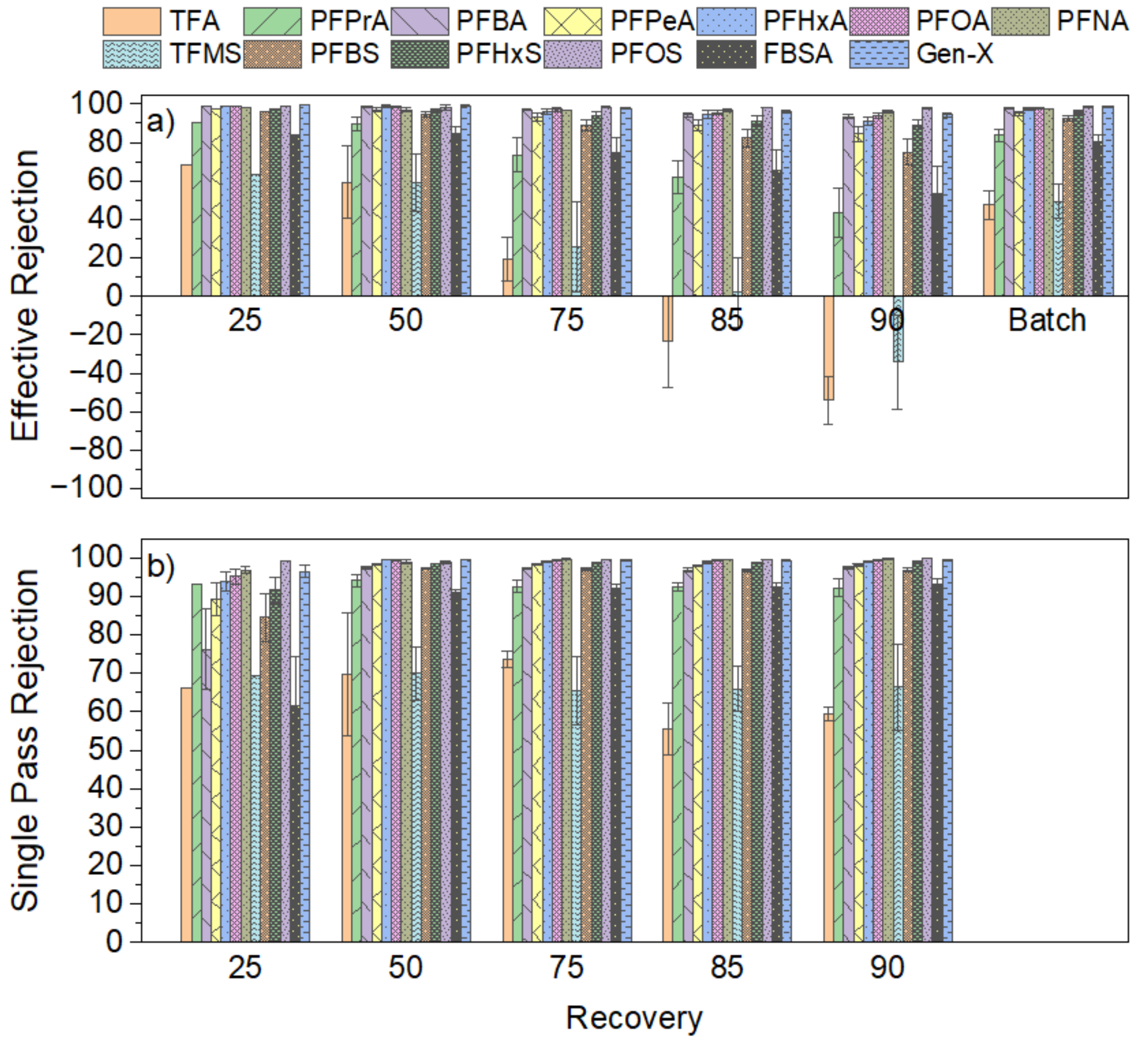

Figure S2: Effect of increasing water recovery on (a) effective rejection of PFAS (eq 2) and (b) single pass rejection of PFAS (eq 1) measured in batch experiments conducted on day 1 of continuous membrane operation. Conditions: 25 LMH, 15% single pass recovery, 2540 NF270 membrane module, PFAS feedwater concentrations listed in Table 1. Error bars represent range of values observed in duplicate experiments.

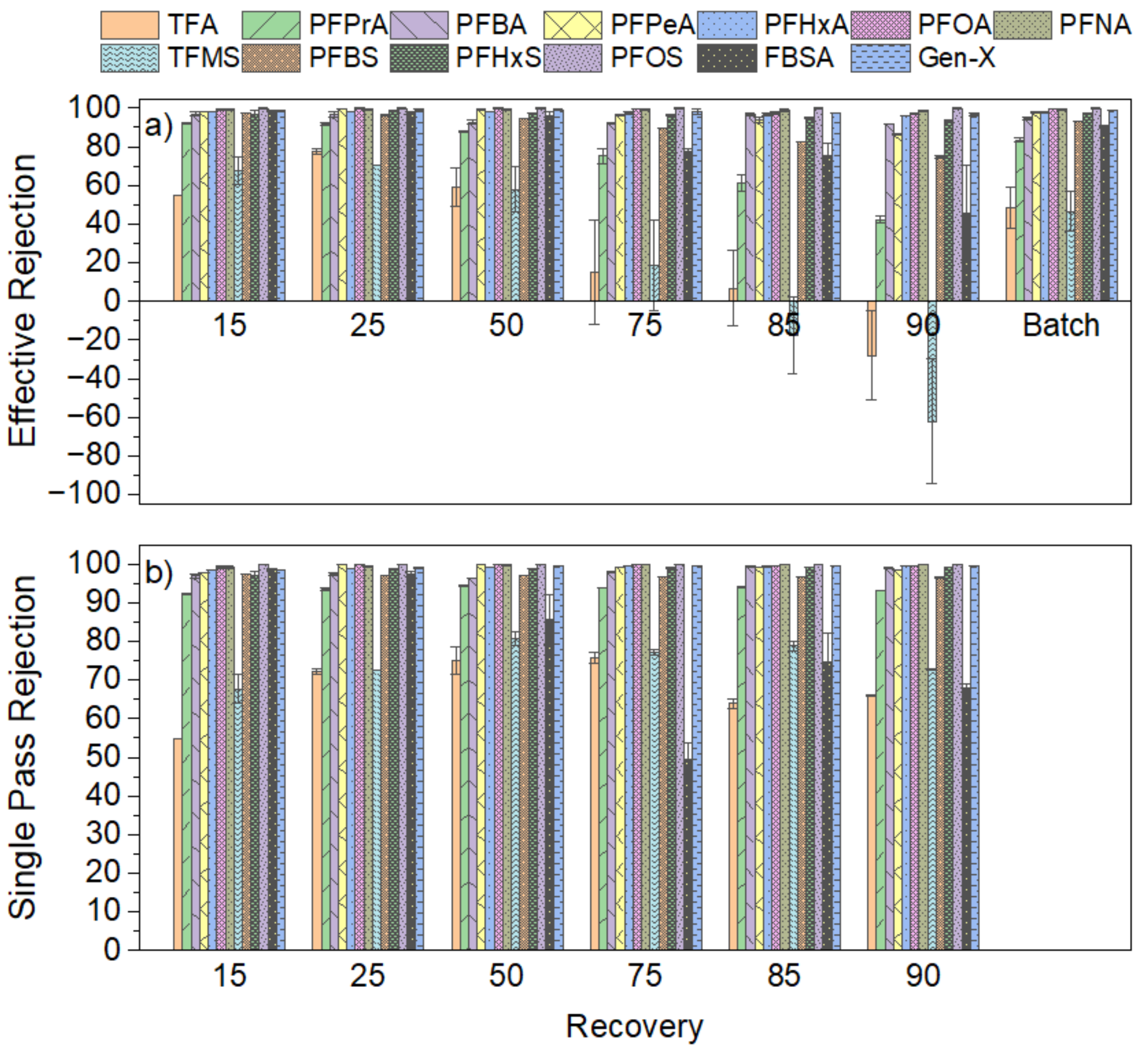

Figure S3: Effect of increasing water recovery on (a) effective rejection of PFAS (eq 2) and (b) single pass rejection of PFAS (eq 1) measured in batch experiments conducted on day 15 of continuous membrane operation. Conditions: 25 LMH, 15% single pass recovery, 2540 NF270 membrane module, PFAS feedwater concentrations listed in Table 1. Error bars represent range of values observed in duplicate experiments.

Section S8: Permeate and feed concentrations for NF experiments.

Table S8: Permeate and feed PFAS concentrations for day 1, 15, and 30 batch experiments.

Day	Location	Recovery	TFA	PFPrA	PFBA	PFPeA	PFHxA	PFOA	PFNA	TFMS	PFBS	PFHxS	PFOS	FBSA	Gen-X
1	Permeate	25	1,810	423	24.6	14.8	6.1	4.7	5.5	111	16.9	10.2	3.8	18.1	5.11
		50	2,330 ± 1,070	460 ± 156	28.9 ± 6.3	16.2 ± 5.3	4.7 ± 3.4	4.8 ± 1.7	8.7 ± 3.1	124 ± 45	21.6 ± 6.1	11.9 ± 2.4	7.3 ± 4.3	17.8 ± 4.3	6.28 ± 2.45
		75	4,630 ± 650	1,170 ± 390	59.7 ± 6.7	34.5 ± 10.6	18.8 ± 6.0	11.5 ± 3.7	9.7 ± 0.7	226 ± 72	45.5 ± 12.4	20.4 ± 5.5	6.4 ± 1.8	28.8 ± 8.9	17.2 ± 3.87
		85	7,070 ± 1,370	1,660 ± 380	111 ± 25	56.0 ± 14.4	26.9 ± 11.0	18.4 ± 4.4	10.4 ± 2.5	298 ± 55	72.6 ± 18.8	31.1 ± 8.4	7.0 ± 0.9	39.3 ± 11.7	27.7 ± 5.1
		90	8,840 ± 720	2,480 ± 560	126 ± 15	79.7 ± 19.0	43.8 ± 10.9	23.8 ± 6.3	11.3 ± 1.9	408 ± 75	102 ± 27	38.4 ± 9.2	7.4 ± 1.0	53.5 ± 16.1	41.6 ± 7.4
	Feed	25	5,340	6,210	845	654	587	542	456	359	550	517	486	141	1,030
		50	8,120 ± 770	7,700 ± 600	1,090 ± 40	995 ± 34	915 ± 86	873 ± 3	645 ± 66	399 ± 60	772 ± 60	773 ± 23	702 ± 13	195 ± 19	1,550 ± 50
		75	17,400 ± 1,000	15,300 ± 1,700	2,050 ± 40	1,980 ± 40	1,590 ± 40	1,700 ± 30	1,250 ± 40	641 ± 42	1,510 ± 30	1,490 ± 10	1,390 ± 30	361 ± 24	2,890 ± 20
		85	15,800 ± 700	21,600 ± 2,300	3,550 ± 430	2,860 ± 70	2,280 ± 80	2,480 ± 60	1,980 ± 100	872 ± 14	2,190 ± 130	2,110 ± 40	1,990 ± 100	519 ± 39	4,230 ± 40
		90	21,700 ± 800	32,300 ± 2,300	5,330 ± 1,000	4,680 ± 530	4,720 ± 550	3,770 ± 300	3,100 ± 400	1,290 ± 200	3,370 ± 360	3,360 ± 310	4,670 ± 1,840	808 ± 67	6,640 ± 720
15	Permeate	15	5,730 ± 0	280 ± 0	14.0 ± 2.9	6.7 ± 0.6	6.7 ± 0	2.5 ± 1.9	3.8 ± 2.5	82.5 ± 3.4	9.7 ± 0.0	10.9 ± 6.7	0.6 ± 0.5	24.2 ± 3.2	8.6 ± 0.1
		25	2,450 ± 270	310 ± 46	15.9 ± 4.9	0.9 ± 0.2	6.7 ± 0	0.2 ± 0.2	4.0 ± 2.2	92.1 ± 0.0	13.6 ± 1.1	6.6 ± 1.6	0.7 ± 0.6	27.7 ± 0.7	8.4 ± 4.7
		50	4,320 ± 370	445 ± 7	34.9 ± 0.4	1.7 ± 1.0	6.7 ± 0	0.3 ± 0.2	3.4 ± 2.1	106 ± 11	19.6 ± 0.3	8.9 ± 0.4	1.0 ± 1.0	26.6 ± 3.6	4.9 ± 1.7
		75	8,810 ± 1450	902 ± 95	37.4 ± 7.4	11.3 ± 0.6	9.7 ± 3.1	2.0 ± 0.4	3.5 ± 1.9	206 ± 22	39.3 ± 1.1	13.4 ± 2.1	1.2 ± 1.1	41.8 ± 1.9	10.5 ± 6.8
		85	9,840 ± 520	1,410 ± 70	16.7 ± 5.9	19.9 ± 2.3	12.7 ± 1.7	9.0 ± 2.2	6.2 ± 3.4	304 ± 4	65.8 ± 0.2	18.1 ± 0.2	1.0 ± 0.9	63.6 ± 2.3	14.9 ± 0.8
		90	13,600 ± 300	2,120 ± 60	39.6 ± 5.4	42.7 ± 4.0	15.9 ± 0.6	9.7 ± 2.1	6.6 ± 1.0	416 ± 8	96.5 ± 3.7	22.5 ± 2.1	1.0 ± 1.0	91.5 ± 9.7	20.7 ± 5.1
	Feed	15	10,900 ± 1,800	3,670 ± 210	481 ± 66	318 ± 37	403 ± 15	325 ± 2	484 ± 3	266 ± 49	383 ± 3	363 ± 10	346 ± 22	184 ± 44	600 ± 28
		25	8,770 ± 450	4,820 ± 200	593 ± 4	430 ± 11	532 ± 11	453 ± 21	635 ± 69	345 ± 7	452 ± 11	516 ± 29	416 ± 6	225 ± 42	854 ± 41
		50	18,300 ± 3,700	7,890 ± 420	952 ± 66	747 ± 14	889 ± 79	621 ± 32	948 ± 125	563 ± 48	694 ± 33	764 ± 36	644 ± 12	347 ± 1	1,260 80
		75	37,600 ± 10,300	14,600 ± 900	1,770 ± 21	1,390 ± 110	1,980 ± 140	1,190 ± 10	1,900 ± 120	892 ± 41	1,200 ± 40	1,330 ± 0	1,140 ± 70	496 ± 1	2,170 ± 130
		85	27,500 ± 3,500	23,600 ± 2,200	2,730 ± 250	2,110 ± 70	1,990 ± 120	1,840 ± 30	3,030 ± 170	1,460 ± 150	2,000 ± 80	2,090 ± 30	1,590 ± 70	694 ± 15	3,190 ± 80
		90	39,800 ± 1,200	31,500 ± 1,100	3,790 ± 230	2,950 ± 50	3,260 ± 260	2,610 ± 150	3,730 ± 100	1,530 ± 40	2,810 ± 110	2,940 ± 20	2,220 ± 10	894 ± 106	4,930 ± 380
30	Permeate	15	1,090 ± 180	275 ± 3	0.1 ± 0	5.1 ± 1.2	6.2 ± 2.1	5.4 ± 3.0	2.1 ± 2.0	70.6 ± 0.7	7.7 ± 0.5	11.3 ± 8.2	0.6 ± 0.3	47.0 ± 1.8	1.33 ± 0
		25	1,500 ± 40	380 ± 3	0.1 ± 0	4.8 ± 0.3	8.9 ± 2.0	3.7 ± 1.1	2.1 ± 1.7	89.3 ± 6.4	9.7 ± 0.3	6.5 ± 1.4	0.7 ± 0.4	49.9 ± 1.0	1.33 ± 0
		50	2,460 ± 200	626 ± 28	4.3 ± 0.6	14.3 ± 2.7	9.3 ± 1.2	5.1 ± 0.4	2.3 ± 1.3	126 ± 12	17.2 ± 0.0	8.7 ± 0.8	1.0 ± 0.6	67.2 ± 0.2	3.5 ± 0.3
		75	4,980 ± 300	1,390 ± 50	6.0 ± 2.3	28.6 ± 0.2	16.6 ± 0.3	8.5 ± 0.2	2.2 ± 0.7	248 ± 23	33.9 ± 2.3	13.3 ± 1.0	1.2 ± 0.7	103 ± 4	9.3 ± 1.5
		80	7,200 ± 890	2,390 ± 100	54.2 ± 15.6	52.4 ± 1.7	28.5 ± 3.9	12.1 ± 0.9	3.0 ± 0.6	342 ± 3	63.4 ± 0.5	18.4 ± 0.9	1.3 ± 0.8	164 ± 1	16.5 ± 1.5
		90	10,300 ± 100	3,520 ± 10	106 ± 2	73.9 ± 4.5	47.1 ± 0.1	15.2 ± 1.8	3.1 ± 0.6	462 ± 60	94.1 ± 7.7	23.5 ± 2.7	1.3 ± 0.9	226 ± 11	26.3 ± 0.5
	Feed	15	6,420 ± 880	5,240 ± 90	451 ± 60	426 ± 4	458 ± 23	371 ± 12	247 ± 10	262 ± 12	330 ± 0	354 ± 3	355 ± 6	394 ± 35	684 ± 10
		25	3,000 ± 1,020	6,370 ± 370	591 ± 16	547 ± 25	529 ± 0	537 ± 3	350 ± 2	323 ± 50	385 ± 9	473 ± 3	491 ± 2	460 ± 25	779 ± 12
		50	4,700 ± 350	8,820 ± 100	916 ± 51	848 ± 3	801 ± 26	835 ± 3	558 ± 7	458 ± 19	607 ± 12	782 ± 12	759 ± 52	831 ± 11	1,400 ± 70
		75	8,250 ± 290	17,300 ± 700	1,720 ± 20	1,530 ± 80	1,560 ± 20	1,450 ± 10	953 ± 19	799 ± 47	1,060 ± 10	1,330 ± 50	1,350 ± 30	1,260 ± 70	2,420 ± 60
		80	11,200 ± 200	26,600 ± 2,400	2,580 ± 60	2,390 ± 0	2,510 ± 130	2,150 ± 20	1,640 ± 50	1,210 ± 230	1,620 ± 40	2,030 ± 70	2,000 ± 30	1,780 ± 50	3,600 ± 30
		90	12,800 ± 200	33,700 ± 600	3,920 ± 400	3,580 ± 30	3,340 ± 150	3,050 ± 80	2,110 ± 50	1,420 ± 30	2,450 ± 110	2,940 ± 30	2,690 ± 130	4,430 ± 1900	5,500 ± 200

Section S9: Rapid small scale column testing breakthrough curves, plotted BV10 values, and matrix characterization.

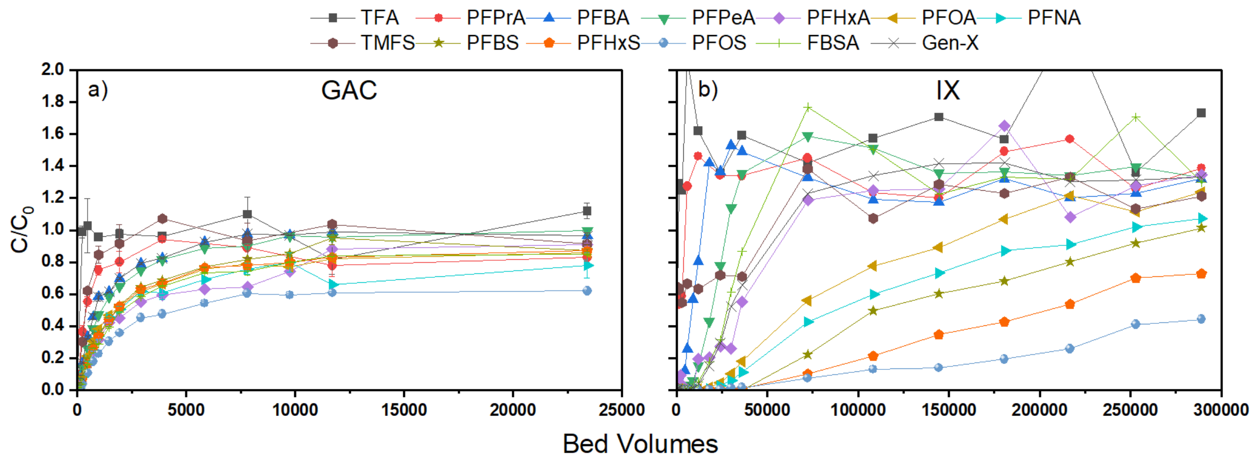

Figure S4: RSSCT breakthrough curves for direct PFAS adsorption from wastewater effluent onto a) GAC and b) IX. GAC conditions: 20 min simulated EBCT, 4 gpm/ft^2 HLR. IX conditions: 2 min simulated EBCT, 9.25 gpm/ft^2 HLR. Error bars represent range of values observed in duplicate experiments.

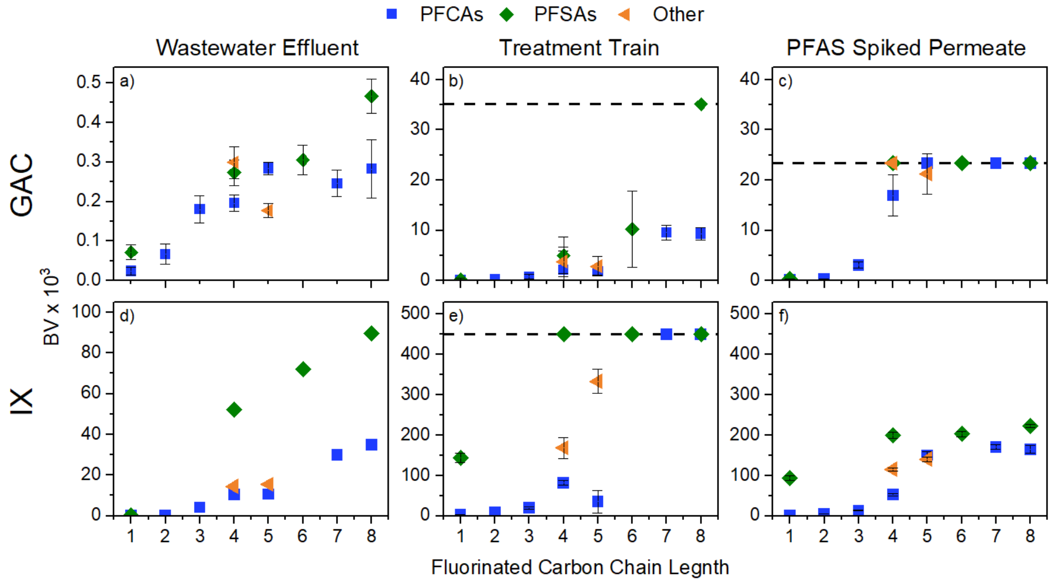

Figure S5: BV$_{10}$ values for GAC and IX for RSSCTs conducted for the direct treatment of wastewater effluent spiked with PFAS, NF-adsorbent treatment train, and PFAS-spiked NF permeate. Dashed lines represent BV of final sampling point, therefore BV$_{10}$ values reported along this line are minimum values.

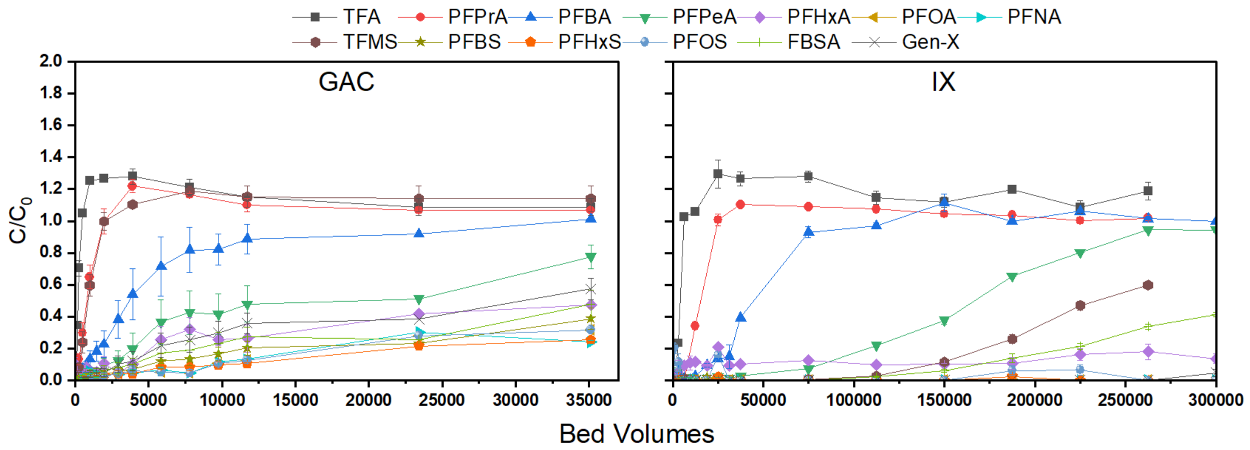

Figure S6: RSSCT breakthrough curves for PFAS adsorption from wastewater effluent spiked with PFAS which was then treated by NF onto a) GAC and b) IX. GAC conditions: 20 min simulated EBCT, 4 gpm/ft^2 HLR. IX conditions: 2 min simulated EBCT, 9.25 gpm/ft^2 HLR. Error bars represent range of values observed in duplicate experiments

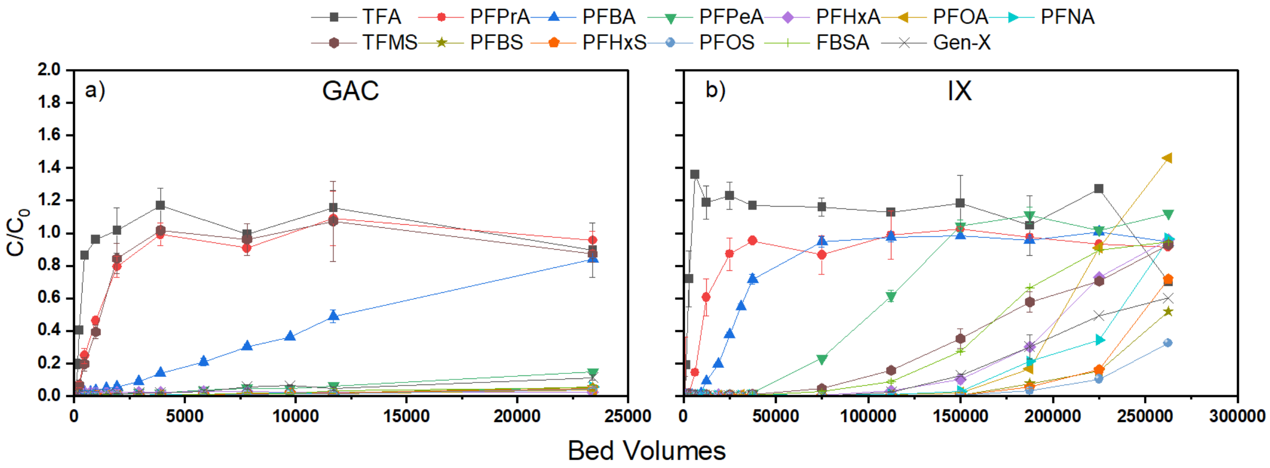

Figure S7: RSSCT breakthrough curves for PFAS adsorption from wastewater effluent treated by the NF system which was then spiked with PFAS onto a) GAC and b) IX. GAC conditions: 20 min simulated EBCT, 4 gpm/ft^2 HLR. IX conditions: 2 min simulated EBCT, 9.25 gpm/ft^2 HLR. Error bars represent range of values observed in duplicate experiments

Table S9: Characteristics of treatment train NF permeate and spiked NF permeate used as RSSCT feed.

Parameter	**Treatment Train** (mg/L)	**Spiked Permeate** (mg/L)
DOC	2.19 ± 0.70	0.90 ± 0.39
TN	5.85 ± 0.15	3.77 ± 0.50
Cl^-	91.7 ± 19.9	79.5 ± 0.4
NO_2^-	3.00 ± 2.01	0.28 ± 0.12
NO_3^-	20.8 ± 4.4	17.2 ± 0.7
PO_4^{3-}	0.59 ± 0.21	2.59 ± 0.21
SO_4^{2-}	1.70 ± 0.18	5.44 ± 1.19
Ca^{2+}	13.8 ± 3.0	10.3 ± 0.1
K^+	9.05 ± 1.63	10.8 ± 0.3
Mg^{2+}	2.71 ± 0.63	2.15 ± 0.05
Na^+	41.3 ±8.8	36.7 ± 0.2
Si	3.20 ±0.44	2.75 ± 0.26

References

(1) Hang, X.; Chen, X.; Luo, J.; Cao, W.; Wan, Y. Removal and Recovery of Perfluorooctanoate from Wastewater by Nanofiltration. *Sep. Purif. Technol.* **2015**, *145*, 120–129. https://doi.org/10.1016/j.seppur.2015.03.013.

(2) Tang, C. Y.; Kwon, Y.-N.; Leckie, J. O. Characterization of Humic Acid Fouled Reverse Osmosis and Nanofiltration Membranes by Transmission Electron Microscopy and Streaming Potential Measurements. *Environ. Sci. Technol.* **2007**, *41* (3), 942–949. https://doi.org/10.1021/es061322r.

(3) López-Muñoz, M. J.; Sotto, A.; Arsuaga, J. M.; Van der Bruggen, B. Influence of Membrane, Solute and Solution Properties on the Retention of Phenolic Compounds in Aqueous Solution by Nanofiltration Membranes. *Sep. Purif. Technol.* **2009**, *66* (1), 194–201. https://doi.org/10.1016/j.seppur.2008.11.001.

(4) Zhu, A.; Long, F.; Wang, X.; Zhu, W.; Ma, J. The Negative Rejection of H+ in NF of Carbonate Solution and Its Influences on Membrane Performance. *Chemosphere* **2007**, *67* (8), 1558–1565. https://doi.org/10.1016/j.chemosphere.2006.11.065.

(5) Boussu, K.; Zhang, Y.; Cocquyt, J.; Van der Meeren, P.; Volodin, A.; Van Haesendonck, C.; Martens, J. A.; Van der Bruggen, B. Characterization of Polymeric Nanofiltration Membranes for Systematic Analysis of Membrane Performance. *J. Membr. Sci.* **2006**, *278* (1), 418–427. https://doi.org/10.1016/j.memsci.2005.11.027.

(6) Cornelis, G.; Boussu, K.; Van Der Bruggen, B.; Devreese, I.; Vandecasteele, C. Nanofiltration of Nonionic Surfactants: Effect of the Molecular Weight Cutoff and Contact Angle on Flux Behavior. *Ind. Eng. Chem. Res.* **2005**, *44* (20), 7652–7658. https://doi.org/10.1021/ie0501226.

(7) Do, V. T.; Tang, C. Y.; Reinhard, M.; Leckie, J. O. Effects of Hypochlorous Acid Exposure on the Rejection of Salt, Polyethylene Glycols, Boron and Arsenic(V) by Nanofiltration and Reverse Osmosis Membranes. *Water Res.* **2012**, *46* (16), 5217–5223. https://doi.org/10.1016/j.watres.2012.06.044.

(8) Tajdini, B.; Vatankhah, H.; Murray, C. C.; Liethen, A.; Bellona, C. Impact of Effluent Organic Matter on Perfluoroalkyl Acid Removal from Wastewater Effluent by Granular Activated Carbon and Alternative Adsorbents. *Water Res.* **2023**, *241*, 120105. https://doi.org/10.1016/j.watres.2023.120105.

(9) Murray, C. C.; Vatankhah, H.; McDonough, C. A.; Nickerson, A.; Hedtke, T. T.; Cath, T. Y.; Higgins, C. P.; Bellona, C. L. Removal of Per- and Polyfluoroalkyl Substances Using Super-Fine Powder Activated Carbon and Ceramic Membrane Filtration. *J. Hazard. Mater.* **2019**, *366*, 160–168. https://doi.org/10.1016/j.jhazmat.2018.11.050.

(10) Zhang, C.; Hao, S.; Gonda, N.; Zhi, Y.; Strathmann, T. J.; Schaefer, C. E.; Higgins, C. P. Quantification of Long–Chain, Short–Chain, and Ultrashort–Chain Liquid Chromatography–Amenable PFASs in Water: Evaluation of Approaches and Tradeoffs for AFFF–Impacted Water. *J. Hazard. Mater.* **2024**, *466*, 133591. https://doi.org/10.1016/j.jhazmat.2024.133591.
